# Supplementary figures and images for: Consistency and differences between centrality measures across distinct classes of networks
Source: PLoS One. 2019 Jul 26;14(7):e0220061. doi: 10.1371/journal.pone.0220061 (PMC6660088; doi:10.1371/journal.pone.0220061)

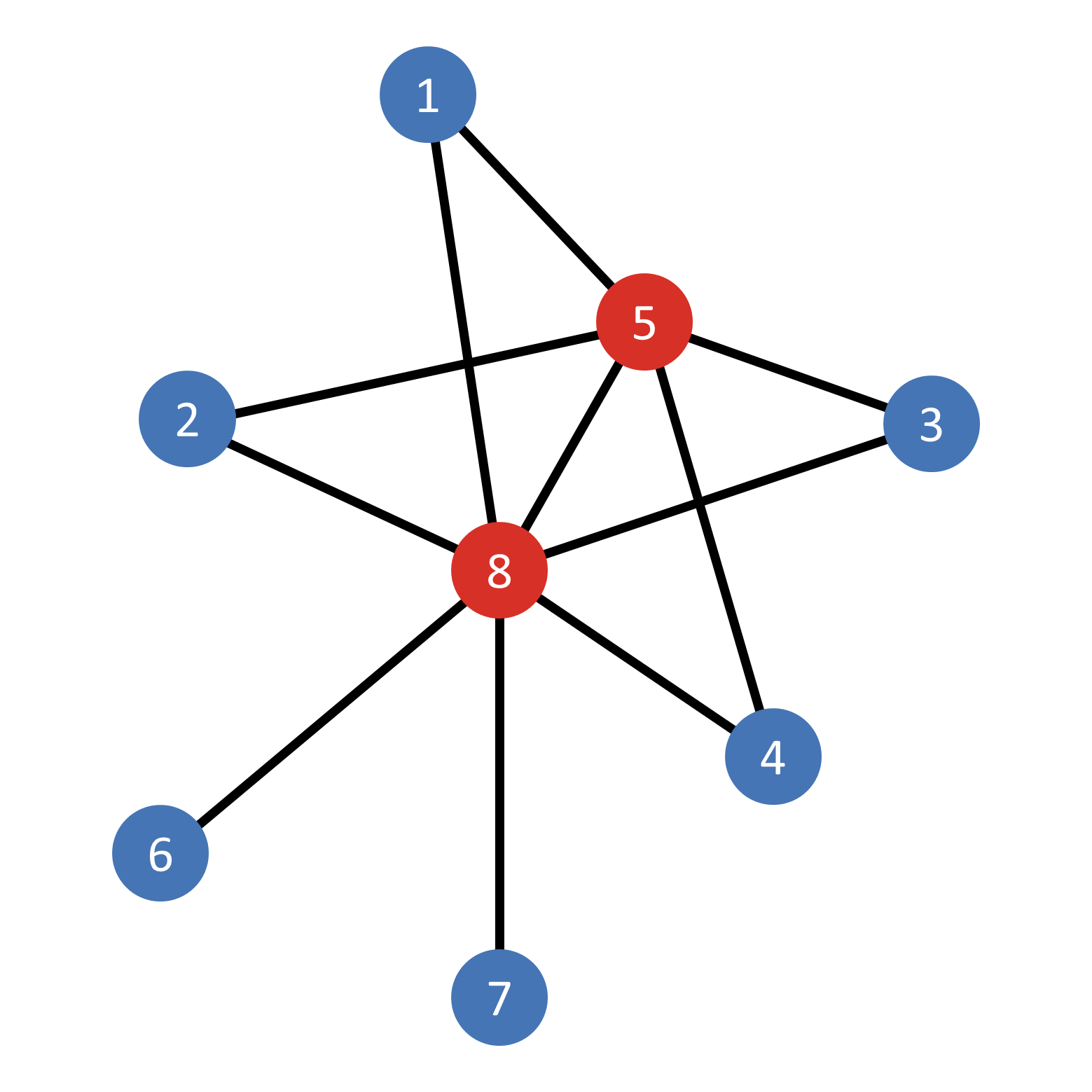

Supplement: S1 Fig — A threshold graph is formed by adding in nodes one at a time in one of two ways: a node can either be added in forming no connections (blue nodes) or a node can be added forming connections to all existing nodes (red nodes). The number in each node is the order in which it was added into the network. (TIF) [file pone.0220061.s001.tif]

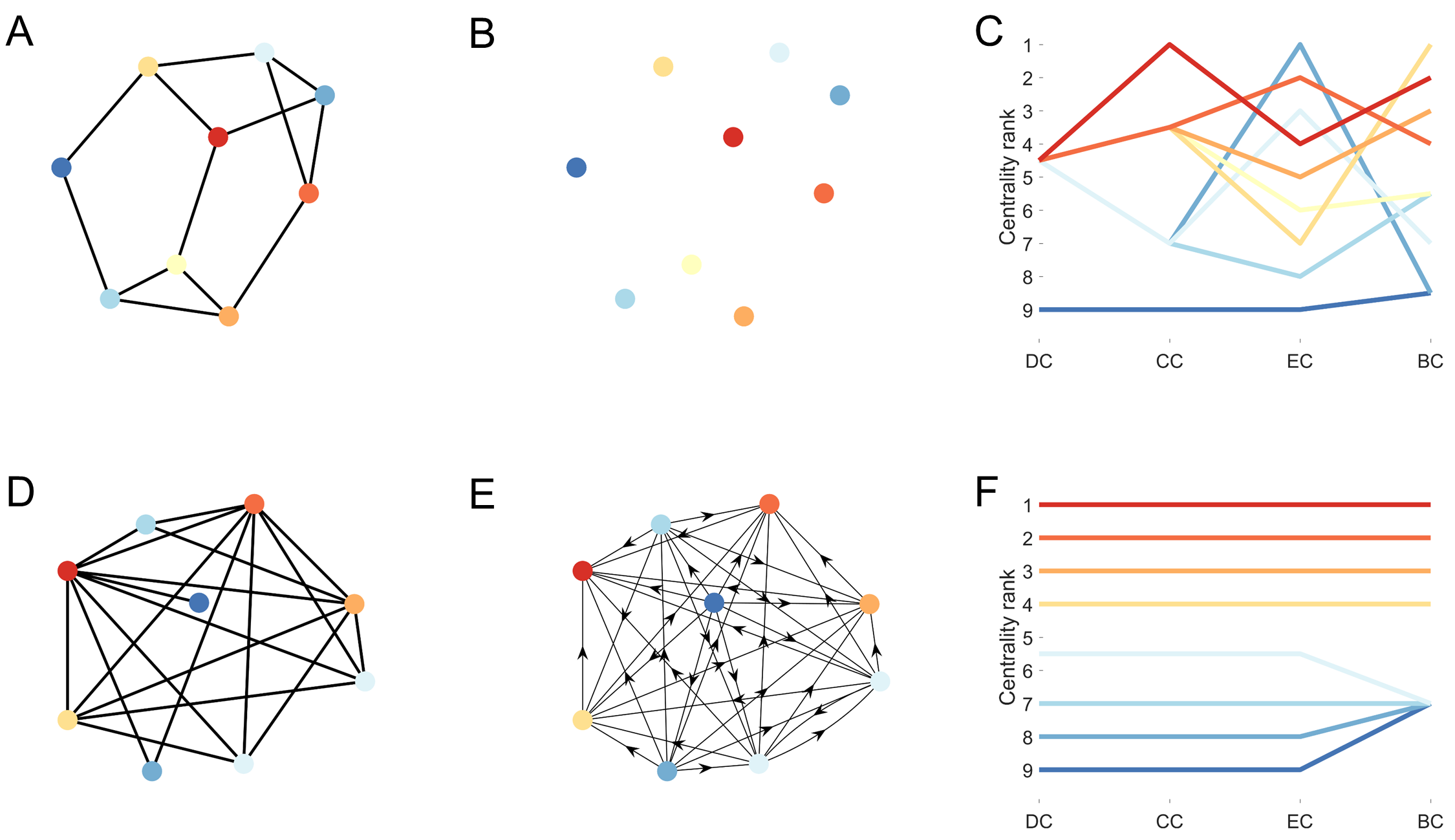

Supplement: S2 Fig — Panels A and D shows a network which demonstrates the neighbourhood-inclusion pre-order and panels B and E shows the dominance relation between nodes (a directed edge indicates that the source node is dominated by the target, that is all the neighbours of the source node are a subset of the neighbours of the target node) for the respective network. The network in panel A has no dominance relationship thus has an inconsistent ranking of nodes by four different centrality measures: degree (DC), closeness (CC), eigenvector (EC), and betweenness (BC), as shown in panel C. Conversely the network in panel D has a complete neighbourhood-inclusion pre-order (each node either dominates or is dominated any other node in the network) and thus all centrality measures will give the same ranking to a node, as shown in panel F. Note that the network in panel D is a threshold graph. A similar figure is presented in Schoch and Brandes [18]. (TIF) [file pone.0220061.s002.tif]

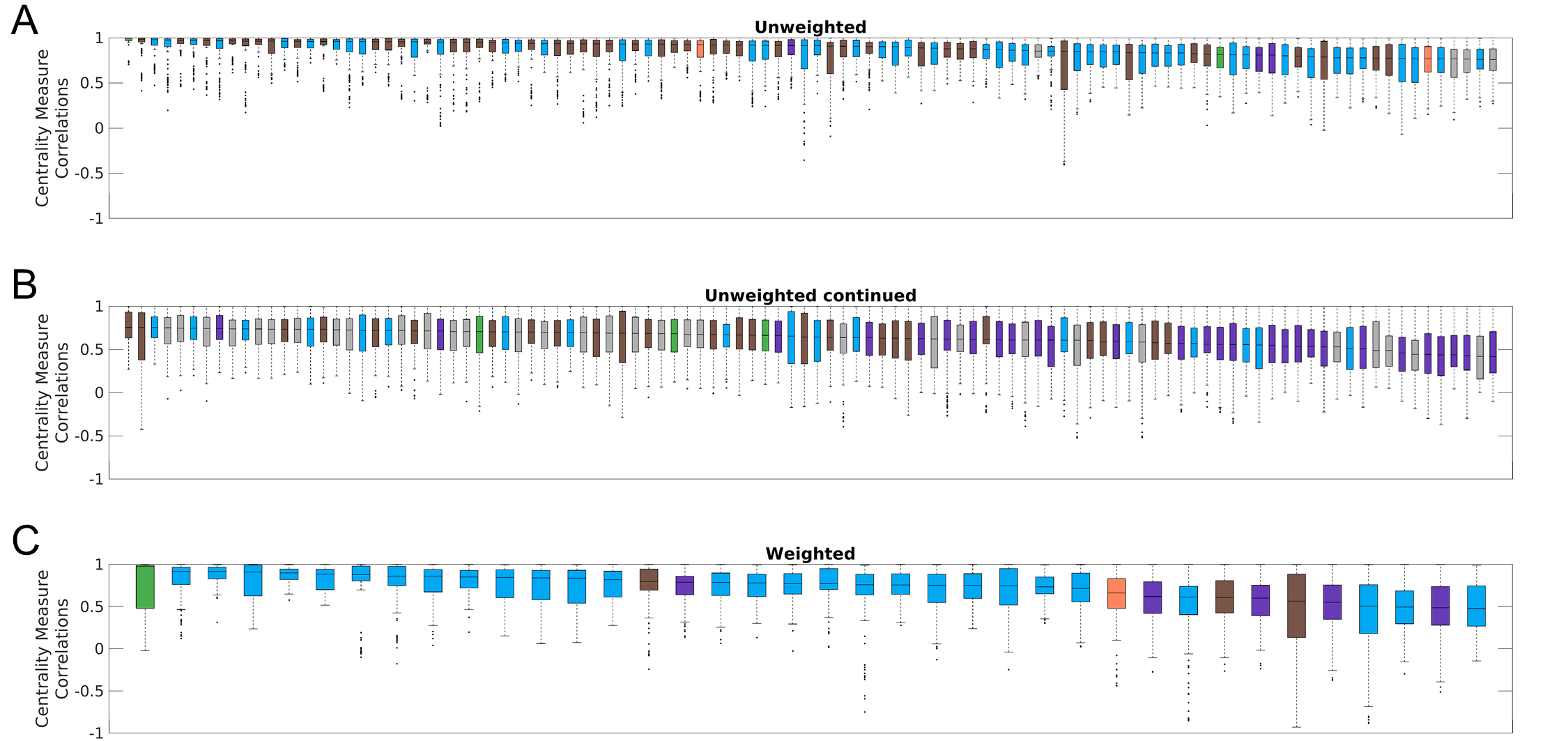

Supplement: S3 Fig — The distribution of Spearman correlation coefficient between every pair of centrality measures, CMCs, in each network is represented as a boxplot. Distributions for unweighted networks are split across panels A and B, while distributions for weighted networks are shown in panel C. Networks are coloured by their natural category (blue = social, grey = technological, brown = biological, orange = informational, purple = transportation; green = economic). Networks have been ordered from highest (left) to lowest (right) median CMC. (TIF) [file pone.0220061.s003.tif]

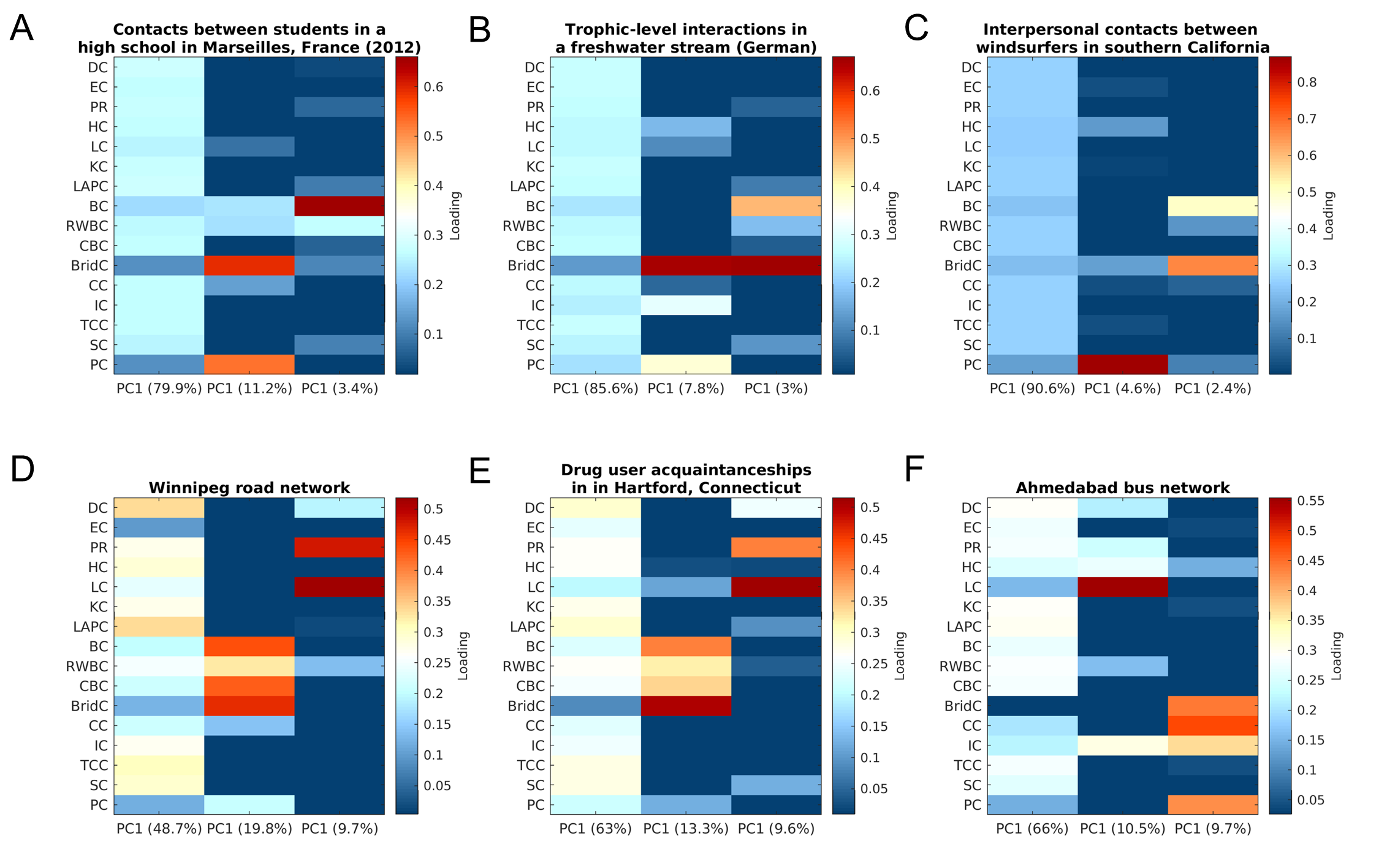

Supplement: S4 Fig — A PCA was run on the (z-scored) centrality scores for each node in each network. The proportion of variance explained by each component is given in the brackets. Nearly all centrality measures loaded onto the first component. In PC2 and PC3 we could observe other types of structure including betweenness measures loading together (panel D-E), bridging centrality/participant coefficient loading together/uniquely on a component (panel A-C), PageRank and leverage loading together (panel D-E), and closeness measures loading with bridging centrality and the participation coefficient (panel F). (TIF) [file pone.0220061.s004.tif]

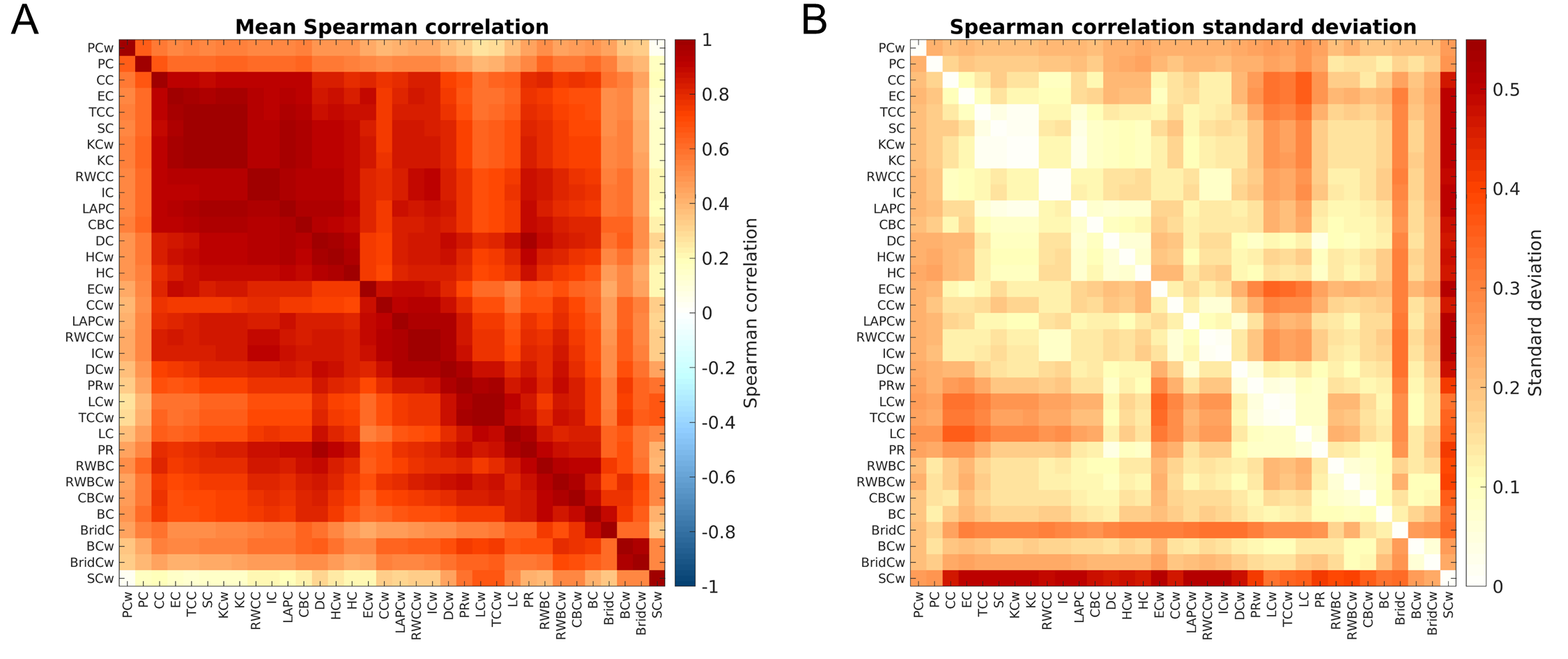

Supplement: S5 Fig — This figure shows the between-network CMC mean (panel A) and standard deviation (panel B) for unweighted and weighted measures, as calculated on the 40 weighted networks. Both weighted and unweighted measures were generally highly correlated with each other, and unweighted measures were more highly intercorrelated than weighted measures. DC = Degree centrality; EC = Eigenvector centrality; KC = Katz centrality; PR = PageRank centrality; LC = Leverage Centrality; HC = H-index centrality; CC = Shortest-path closeness centrality; SC = Subgraph centrality; PC = Participation coefficient; TCC = Total communicability centrality; RWCC = Random-walk closeness centrality; BC = Shortest-path betweenness centrality; CBC = Communicability betweenness centrality; RWBC = Random-walk betweenness centrality; LAPC = Laplacian centrality; BridC = Bridging centrality. A “w” next to the abbreviated name for the centrality measure indicates it is the weighted version. (TIF) [file pone.0220061.s005.tif]

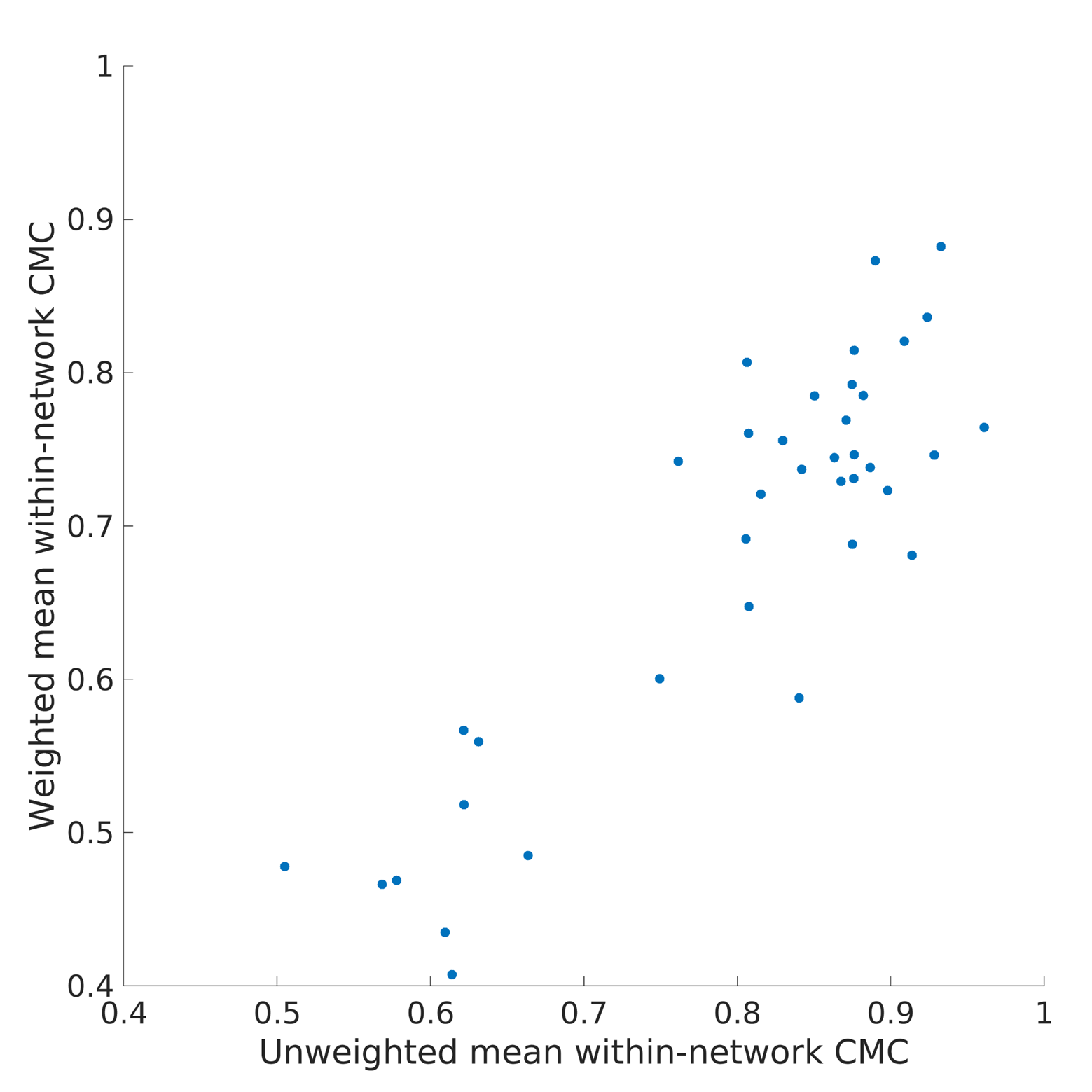

Supplement: S6 Fig — For the 40 networks with edge weights the mean within-network CMC was calculated separately for unweighted and weighted measures, and then these values were plotted against each other. There is a strong relationship between the two indicating higher correlations between unweighted measures is mirrored by higher correlations in weighted measures. (TIF) [file pone.0220061.s006.tif]

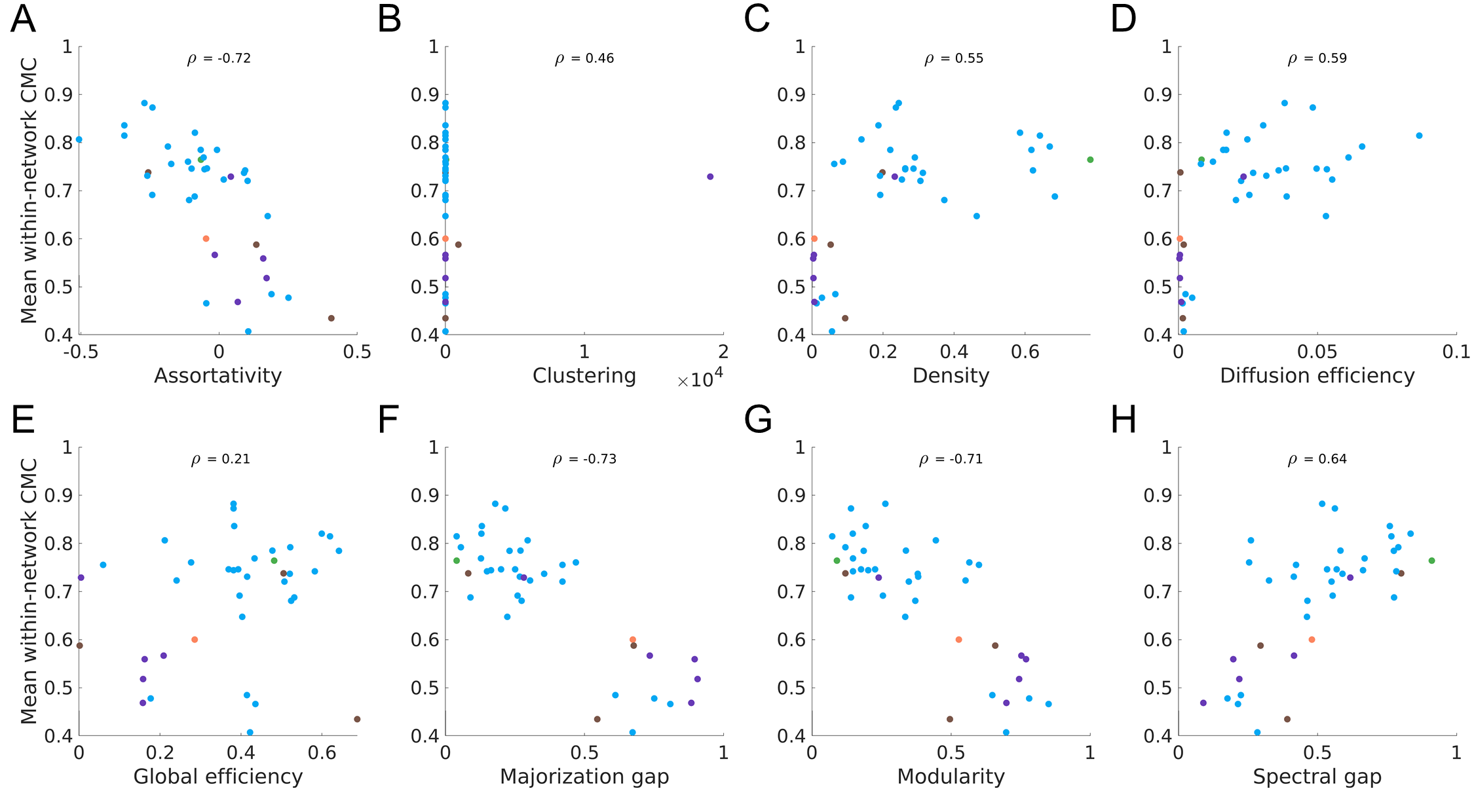

Supplement: S7 Fig — The association between the mean within-network CMC (the average of all CMCs within a single network) and each of the global topological properties. Networks are coloured by their natural category (blue = social, grey = technological, brown = biological, orange = informational, purple = transportation; green = economic). (TIF) [file pone.0220061.s007.tif]

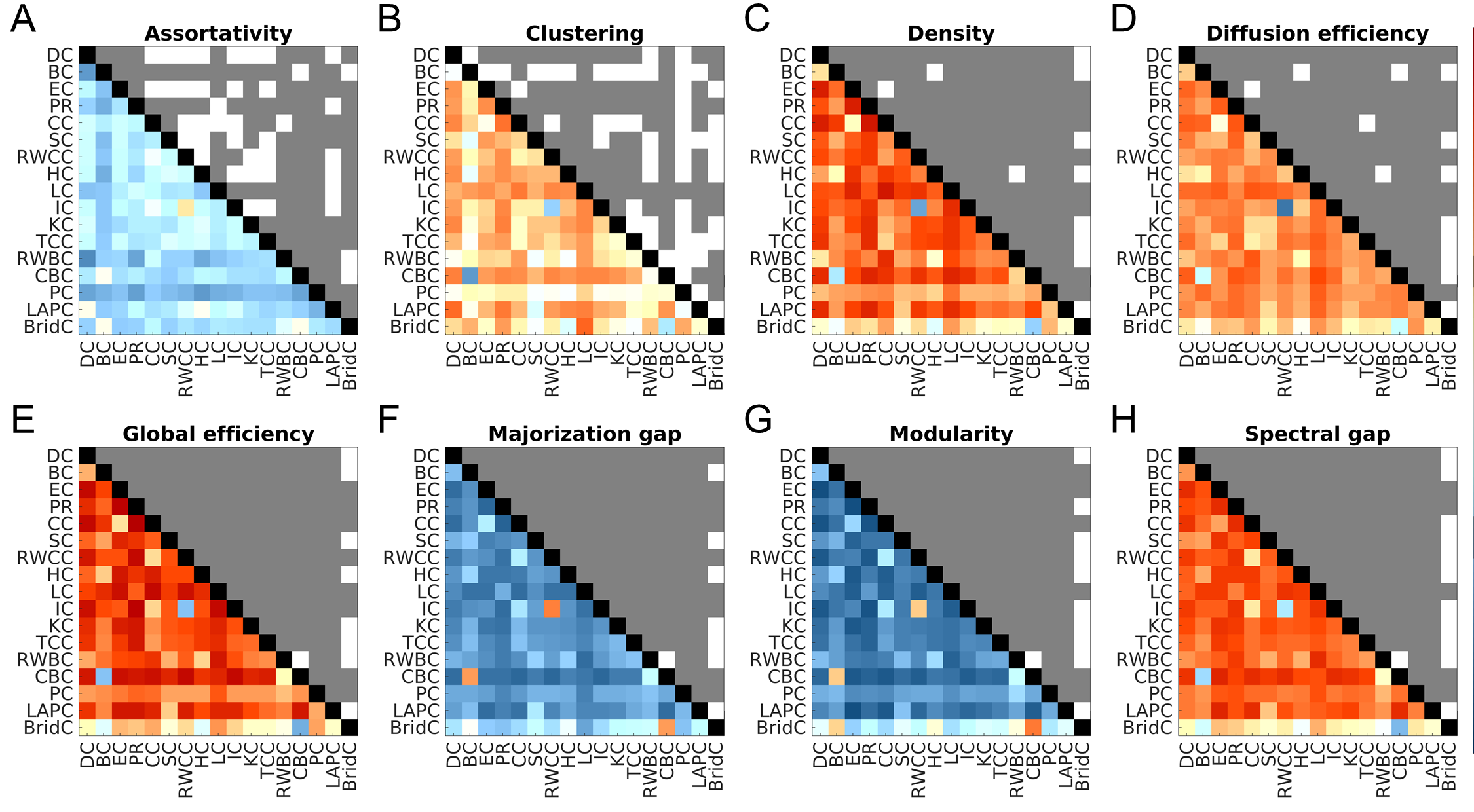

Supplement: S8 Fig — The lower matrix indicates the value of the Spearman correlation between a CMC and a network property. The upper matrix indicates if this correlation was significant (grey) or not (white) when Bonferroni corrected for 136 combinations of centrality measures. This result shows the strength of individual CMCs was correlated with specific network properties. (TIF) [file pone.0220061.s008.tif]

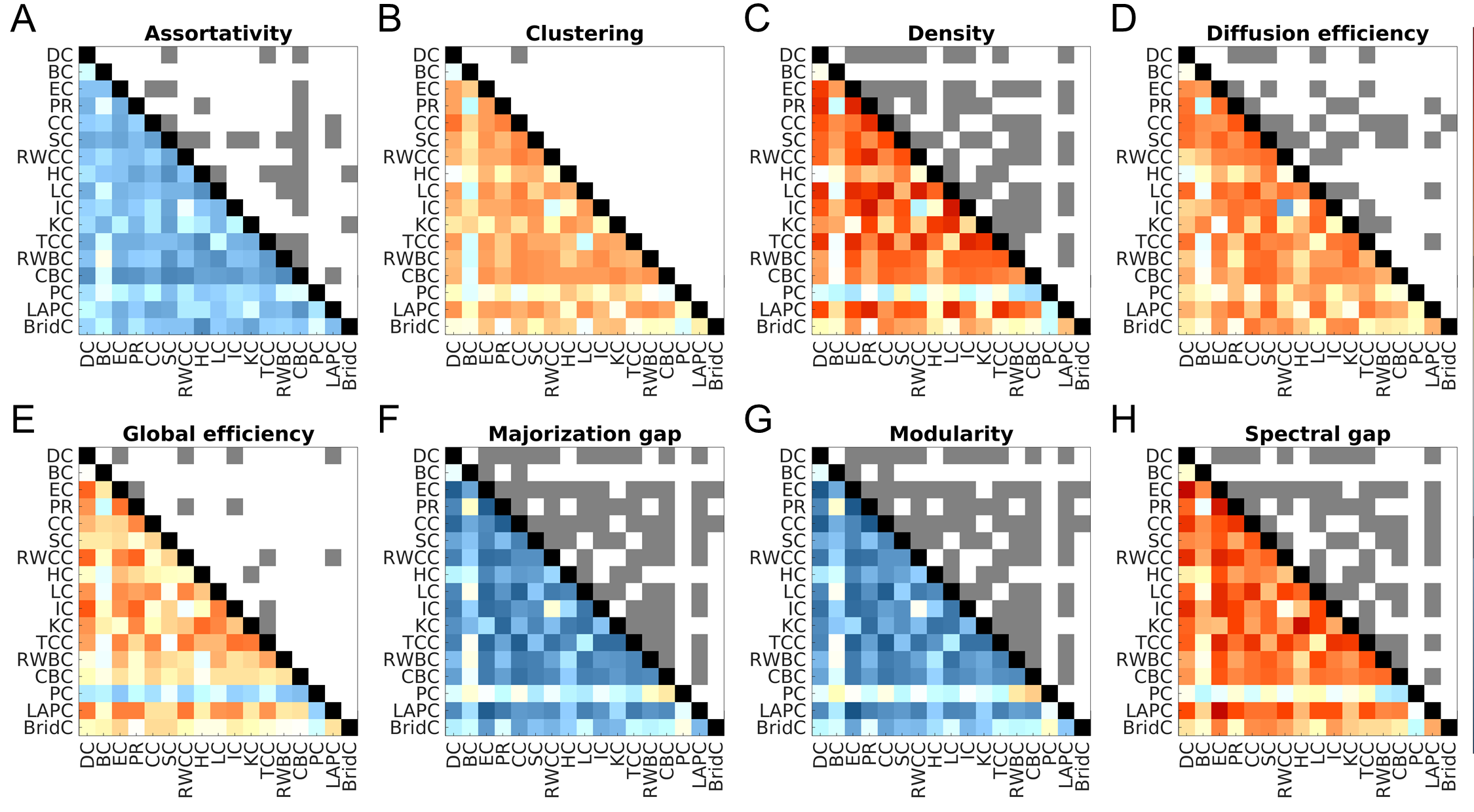

Supplement: S9 Fig — The lower matrix indicates the value of the Spearman correlation between a CMC and a network property. The upper matrix indicates if this correlation was significant (grey) or not (white) when Bonferroni corrected for 136 combinations of centrality measures. This result shows the strength of individual CMCs was correlated with specific network properties. (TIF) [file pone.0220061.s009.tif]

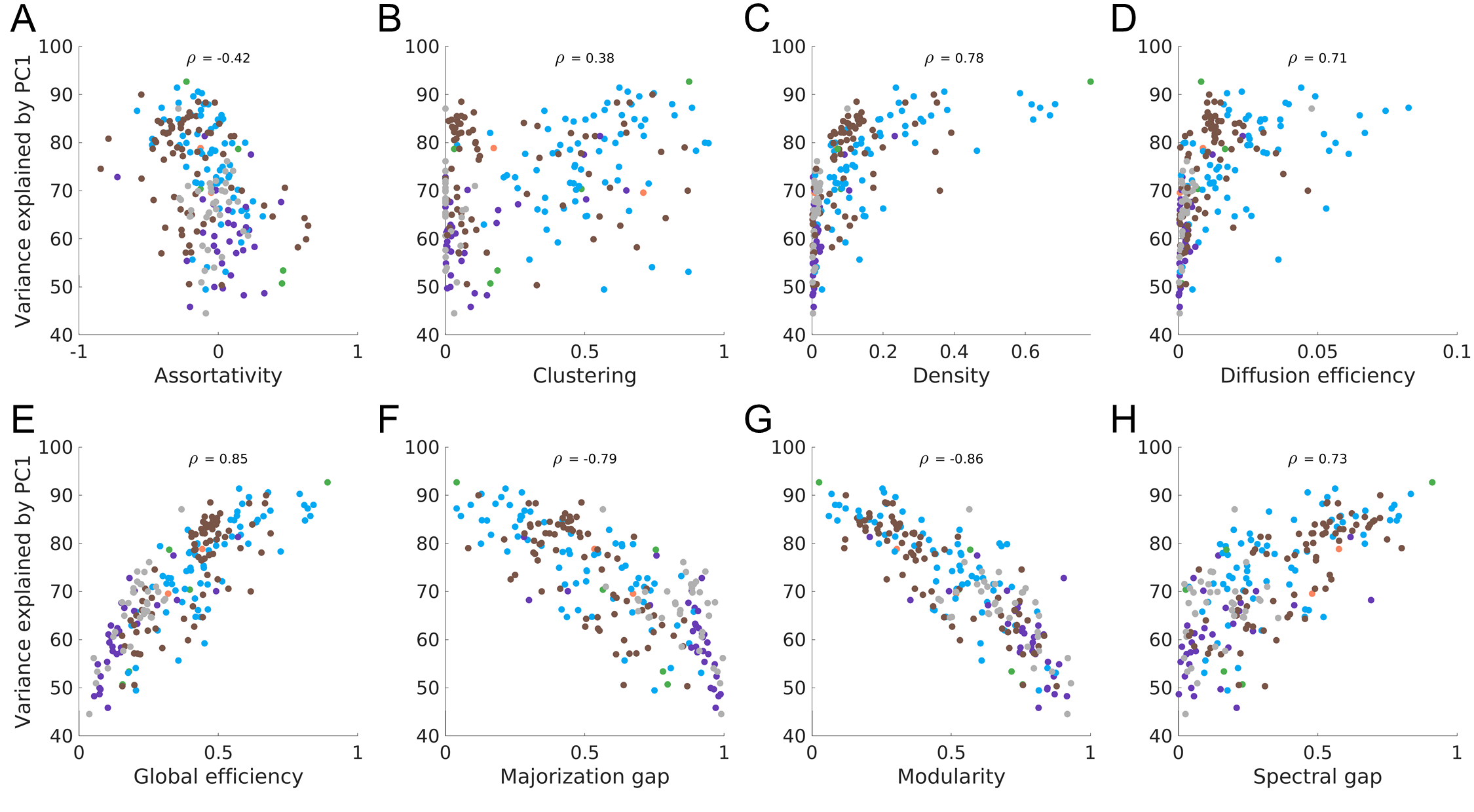

Supplement: S10 Fig — The association between the variance explained by PC1 and each of the global topological properties. Networks are coloured by their natural category (blue = social, grey = technological, brown = biological, orange = informational, purple = transportation; green = economic). (TIF) [file pone.0220061.s010.tif]

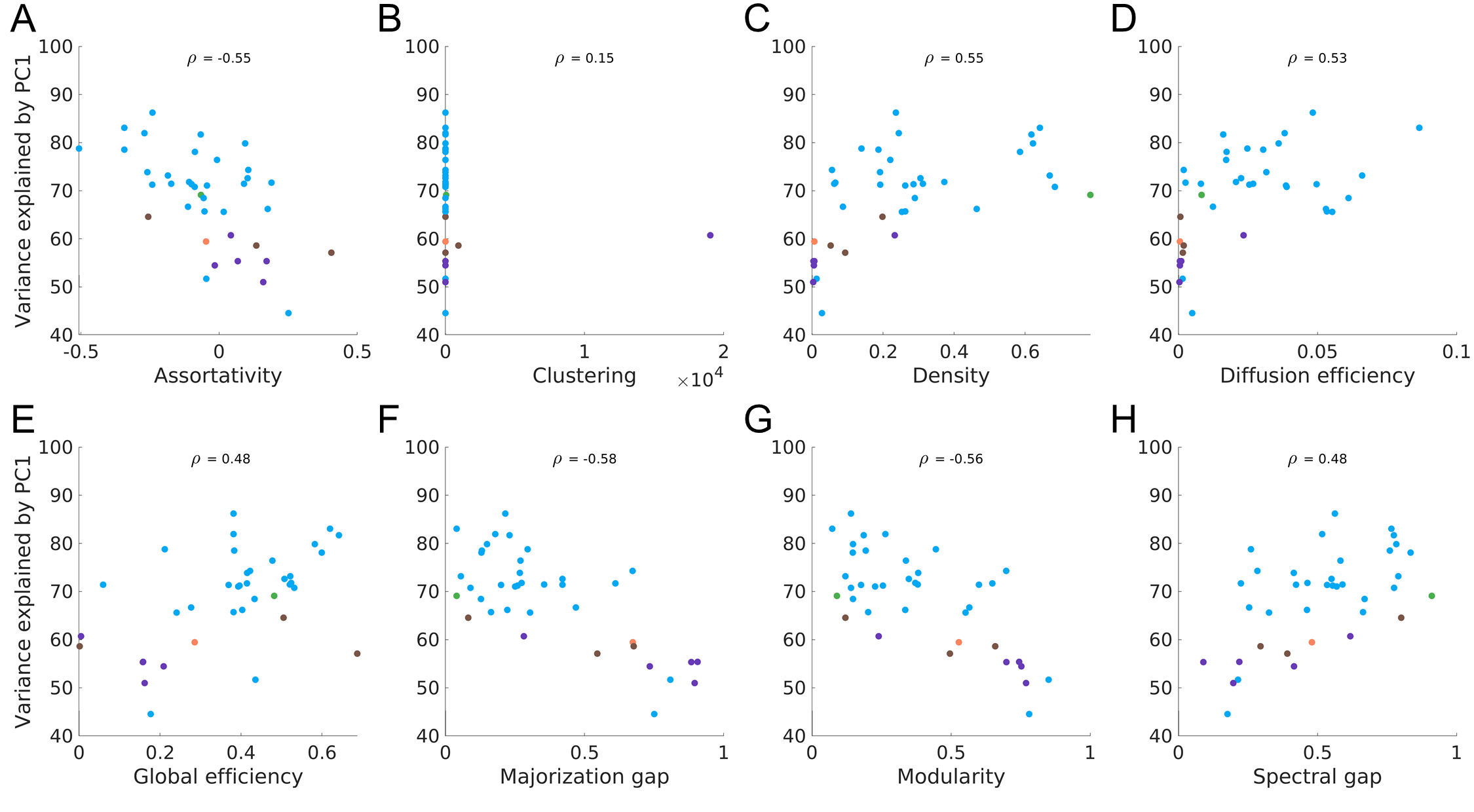

Supplement: S11 Fig — The association between the variance explained by PC1 and each of the global topological properties. Networks are coloured by their natural category (blue = social, grey = technological, brown = biological, orange = informational, purple = transportation; green = economic). (TIF) [file pone.0220061.s011.tif]

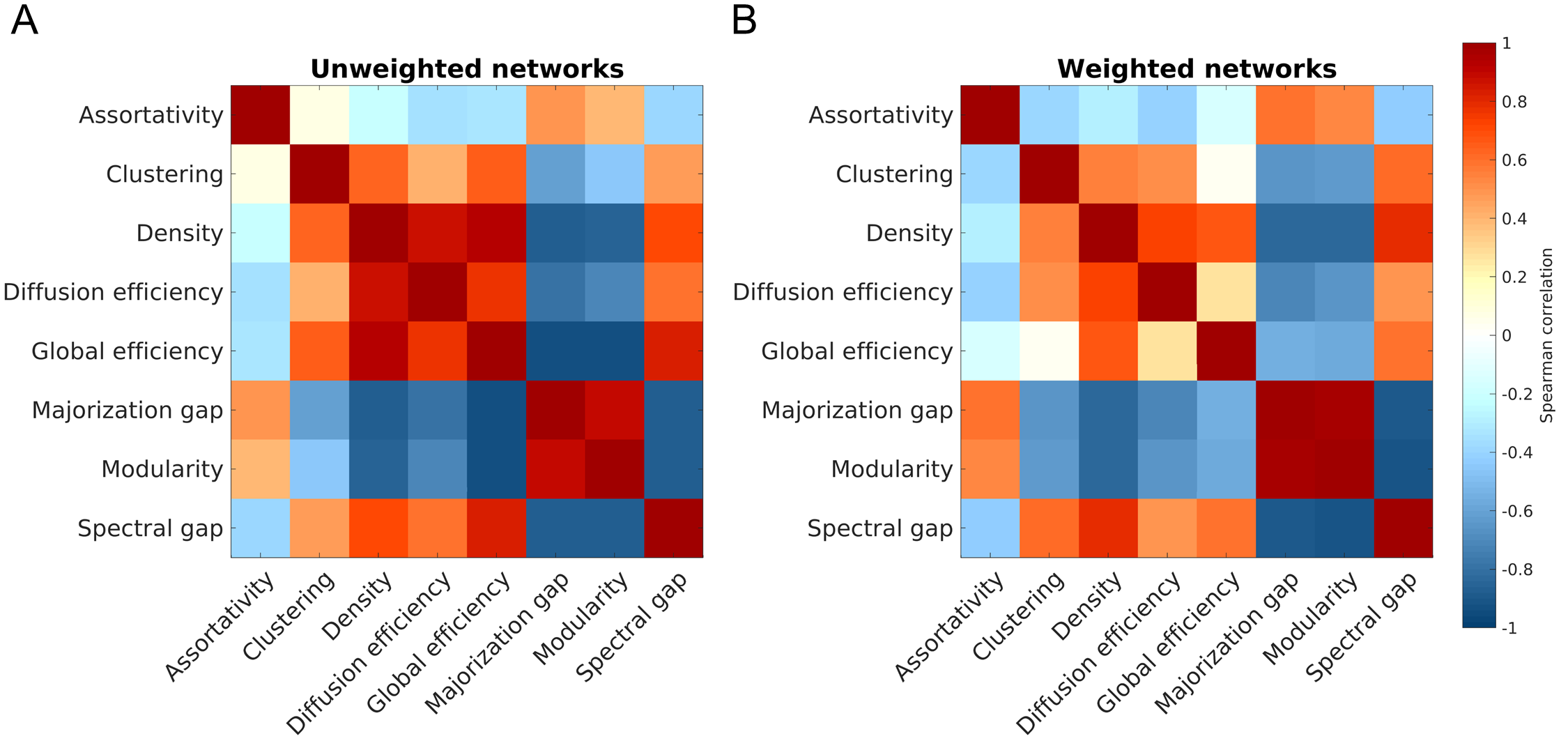

Supplement: S12 Fig — Panel A shows the Spearman correlations between each network property in the unweighted networks, while panel B shows the Spearman correlations between each network property in the weighted networks. (TIF) [file pone.0220061.s012.tif]

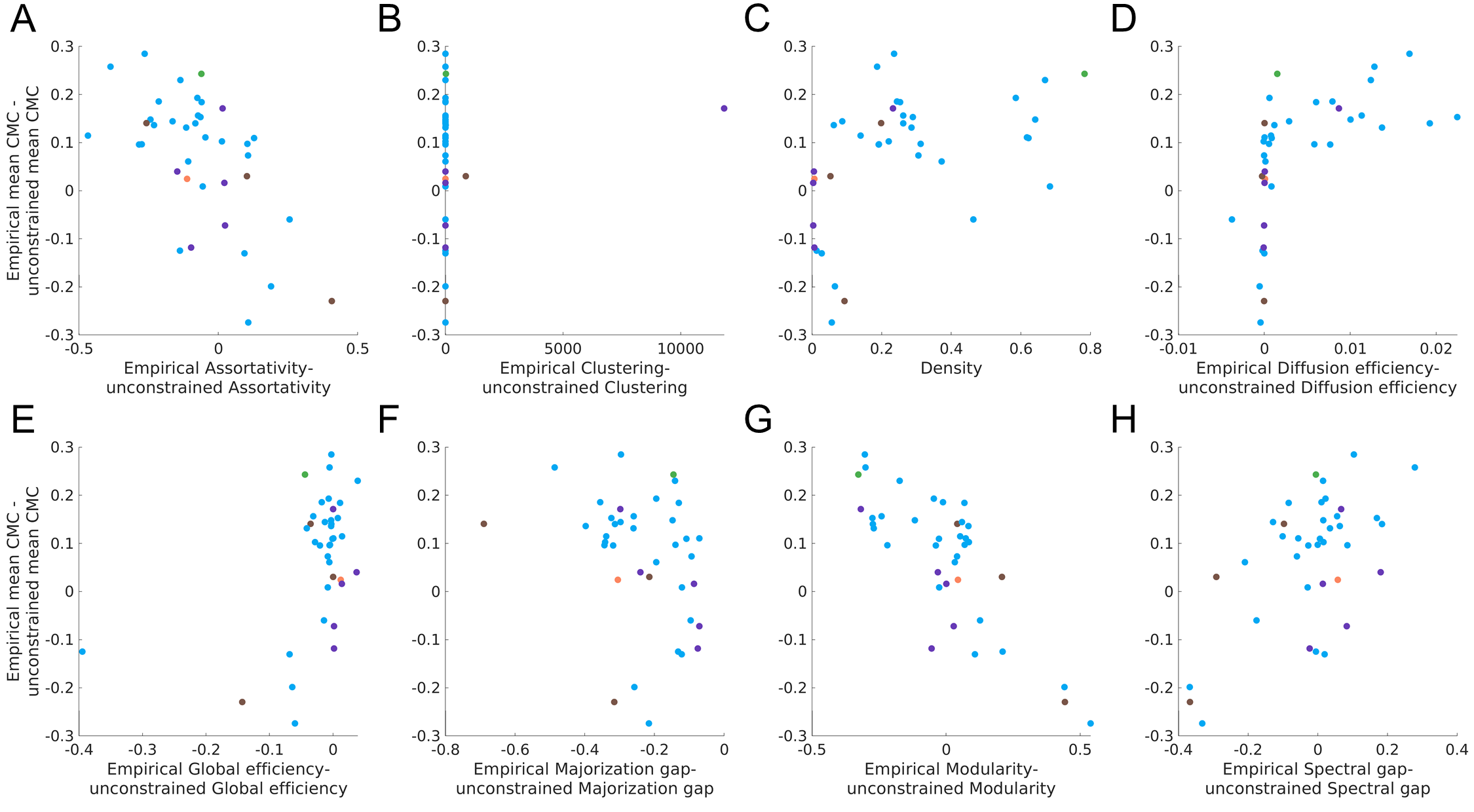

Supplement: S13 Fig — The y-axis of each plot shows the difference between the empirical networks and unconstrained surrogates mean within-network CMC. The x-axis shows the difference between the empirical networks and unconstrained surrogates on a particular property (except for panel C as the unconstrained surrogates have the same density as the empirical network). On both axis, except for the x-axis in panel C, a negative value indicates the empirical network had a lower value than the mean value of the surrogates, while a positive value indicates the empirical networks had a larger value. Points are colored by the natural category of the empirical network (blue = social, grey = technological, brown = biological, orange = informational, purple = transportation; green = economic). (TIF) [file pone.0220061.s013.tif]

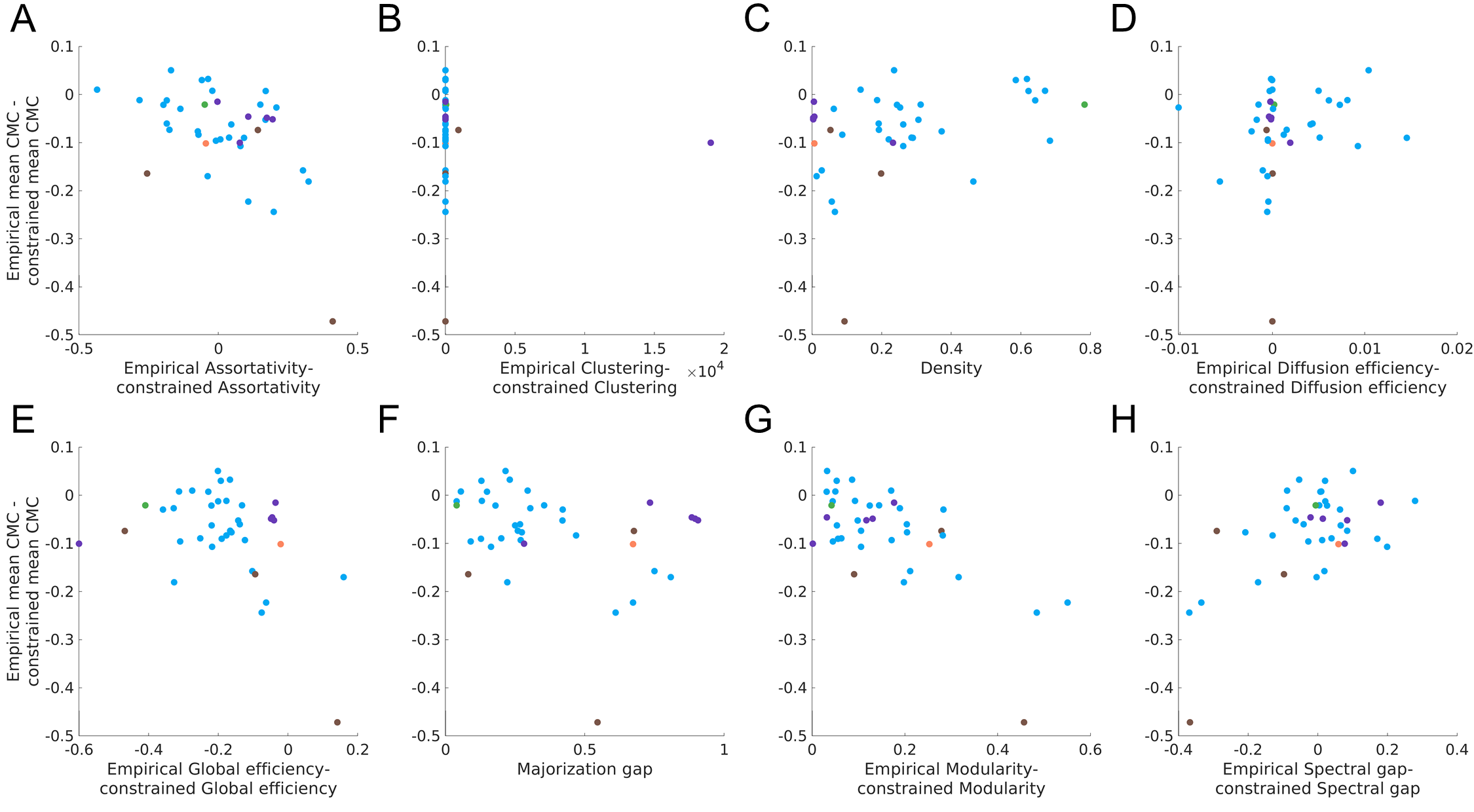

Supplement: S14 Fig — The y-axis of each plot shows the difference between the empirical networks and constrained surrogates mean within-network CMC. The x-axis shows the difference between the empirical networks and constrained surrogates on a particular property (except for panels C and F as the constrained surrogates have the same density and majorization gap as the empirical network). On both axis, except for the x-axis in panels C and F, a negative value indicates the empirical network had a lower value than the mean value of the surrogates, while a positive value indicates the empirical networks had a larger value. Points are colored by the natural category of the empirical network (blue = social, grey = technological, brown = biological, orange = informational, purple = transportation; green = economic). (TIF) [file pone.0220061.s014.tif]

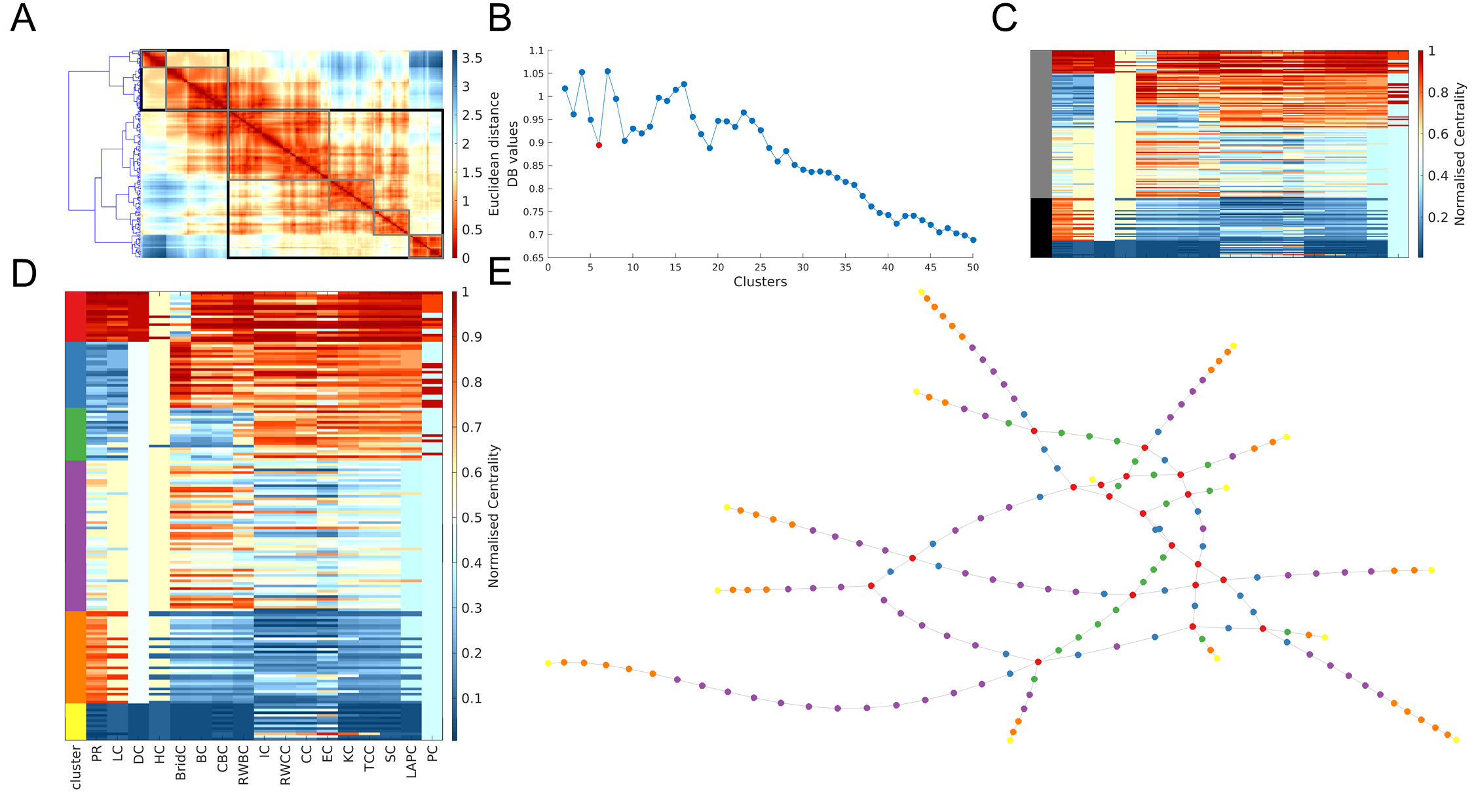

Supplement: S15 Fig — Panel A shows the dendrogram projected alongside the distance matrix of node pairs (ranks scores were normalised to be in the range 0–1 with 1 indicating the highest rank). The black and grey boxes and indicate the clusters when a two-cluster and six-cluster solution is used, respectively. Panel B displays the results for the Davies-Bouldin (DB) criterion. A lower DB value represents a better clustering solution. The solution shown in panels D and E is labelled in red. Only the first 50 clustering solutions are shown for ease of visibility. Panel C shows the matrix of nodal centrality scores (each row is a node and each column is a measure) and how these are clustered in a two-cluster solution (the black and grey represent the two different clusters). Panel D shows the matrix of nodal centrality scores as well as the clusters each node was assigned to. Panel E shows a topological representation of the network, produced using the force-directed layout algorithm, where each node is coloured according to the cluster it was allocated to in panel D. (TIF) [file pone.0220061.s015.tif]

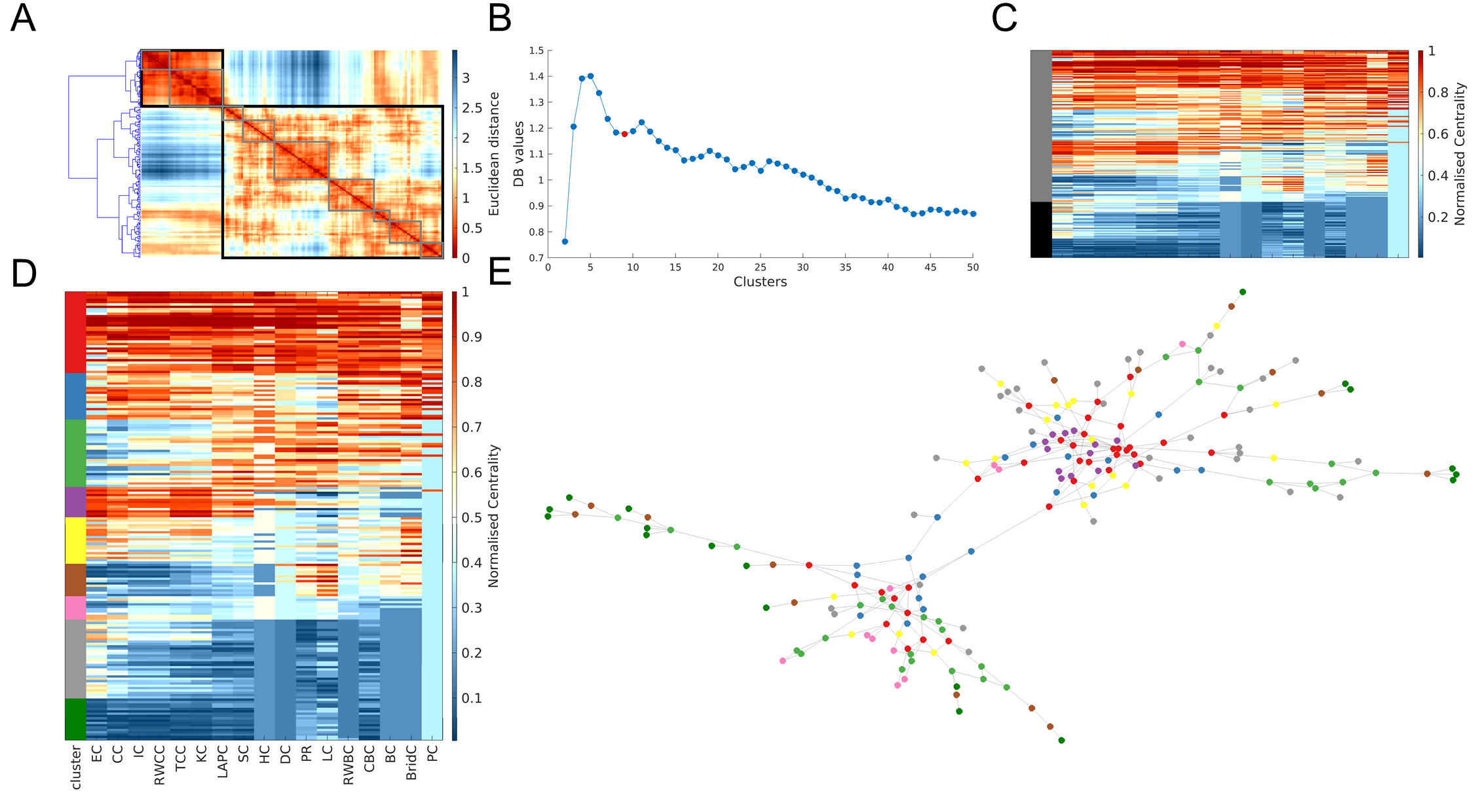

Supplement: S16 Fig — Panel A shows the dendrogram projected alongside the distance matrix of node pairs (ranks scores were normalised to be in the range 0–1 with 1 indicating the highest rank). The black and grey boxes and indicate the clusters when a two-cluster and nine-cluster solution is used, respectively. Panel B displays the results for the Davies-Bouldin (DB) criterion. A lower DB value represents a better clustering solution. The solution shown in panels D and E is labelled in red. Only the first 50 clustering solutions are shown for ease of visibility. Panel C shows the matrix of nodal centrality scores (each row is a node and each column is a measure) and how these are clustered in a two-cluster solution (the black and grey represent the two different clusters). Panel D shows the matrix of nodal centrality scores as well as the clusters each node was assigned to. Panel E shows a topological representation of the network, produced using the force-directed layout algorithm, where each node is coloured according to the cluster it was allocated to in panel D. (TIF) [file pone.0220061.s016.tif]

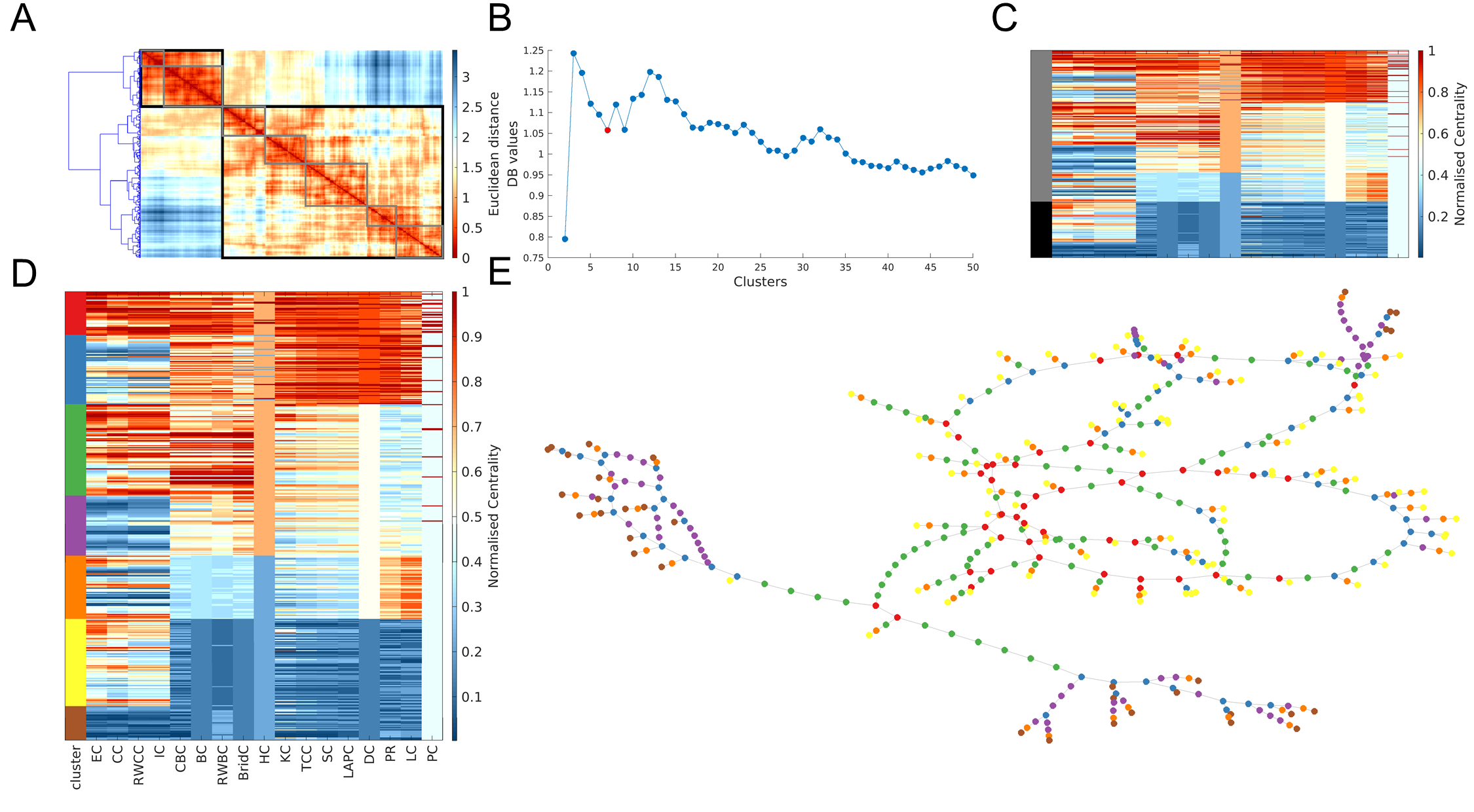

Supplement: S17 Fig — Panel A shows the dendrogram projected alongside the distance matrix of node pairs (ranks scores were normalised to be in the range 0–1 with 1 indicating the highest rank). The black and grey boxes and indicate the clusters when a two-cluster and seven-cluster solution is used, respectively. Panel B displays the results for the Davies-Bouldin (DB) criterion. A lower DB value represents a better clustering solution. The solution shown in panels D and E is labelled in red. Only the first 50 clustering solutions are shown for ease of visibility. Panel C shows the matrix of nodal centrality scores (each row is a node and each column is a measure) and how these are clustered in a two-cluster solution (the black and grey represent the two different clusters). Panel D shows the matrix of nodal centrality scores as well as the clusters each node was assigned to. Panel E shows a topological representation of the network, produced using the force-directed layout algorithm, where each node is coloured according to the cluster it was allocated to in panel D. (TIF) [file pone.0220061.s017.tif]

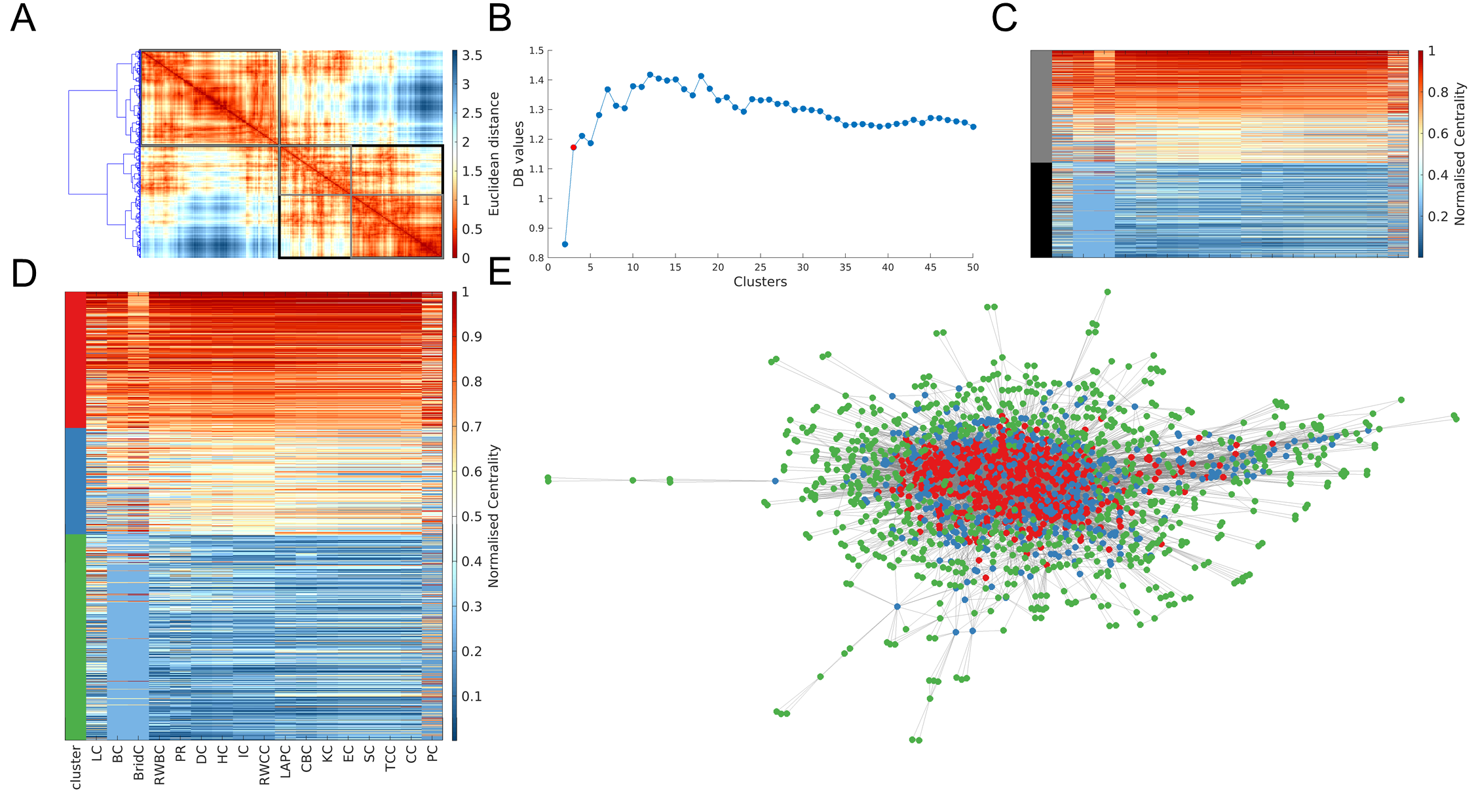

Supplement: S18 Fig — Panel A shows the dendrogram projected alongside the distance matrix of node pairs (ranks scores were normalised to be in the range 0–1 with 1 indicating the highest rank). The black and grey boxes and indicate the clusters when a two-cluster and three-cluster solution is used, respectively. Panel B displays the results for the Davies-Bouldin (DB) criterion. A lower DB value represents a better clustering solution. The solution shown in panels D and E is labelled in red. Only the first 50 clustering solutions are shown for ease of visibility. Panel C shows the matrix of nodal centrality scores (each row is a node and each column is a measure) and how these are clustered in a two-cluster solution (the black and grey represent the two different clusters). Panel D shows the matrix of nodal centrality scores as well as the clusters each node was assigned to. Panel E shows a topological representation of the network, produced using the force-directed layout algorithm, where each node is coloured according to the cluster it was allocated to in panel D. (TIF) [file pone.0220061.s018.tif]

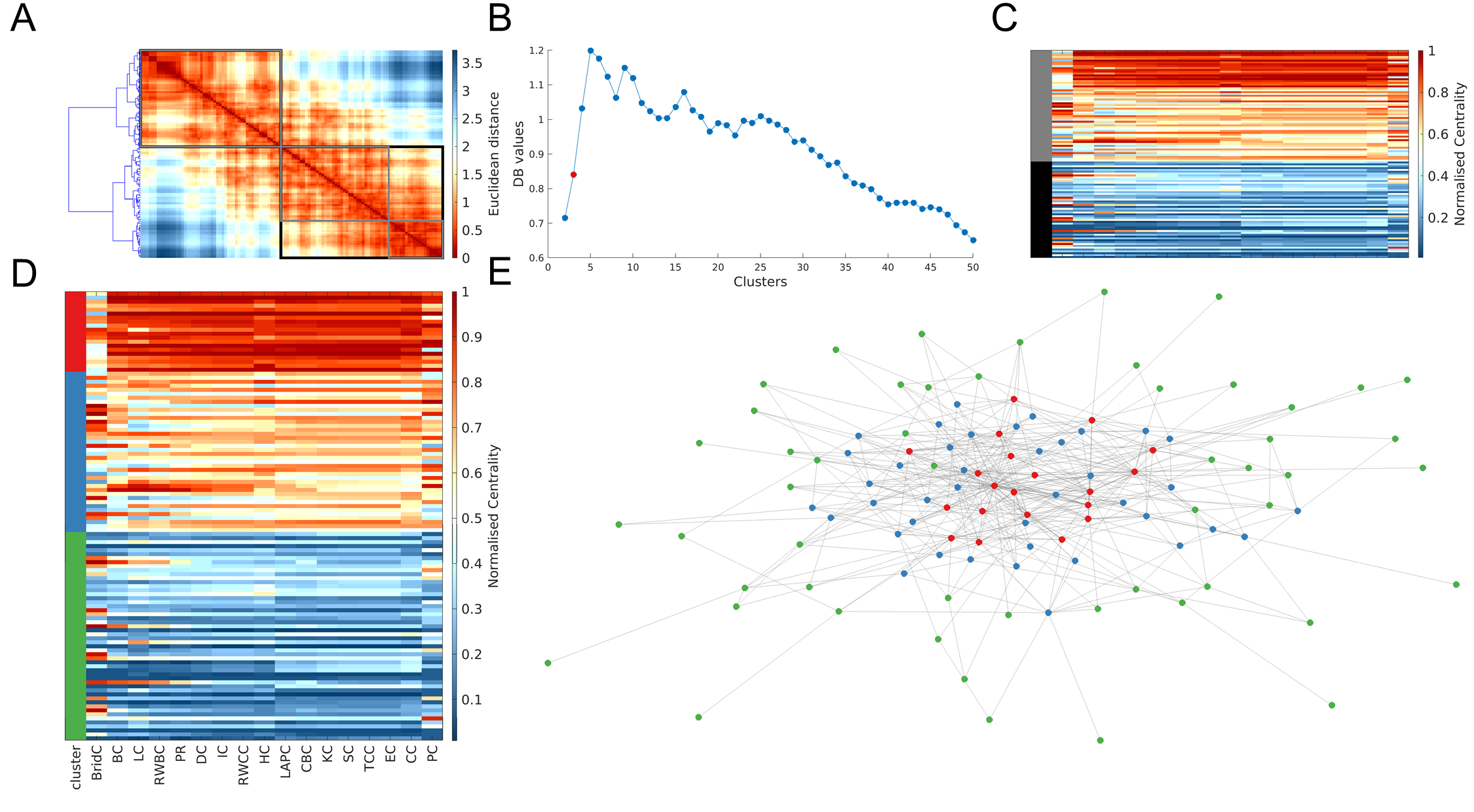

Supplement: S19 Fig — Panel A shows the dendrogram projected alongside the distance matrix of node pairs (ranks scores were normalised to be in the range 0–1 with 1 indicating the highest rank). The black and grey boxes and indicate the clusters when a two-cluster and three-cluster solution is used, respectively. Panel B displays the results for the Davies-Bouldin (DB) criterion. A lower DB value represents a better clustering solution. The solution shown in panels D and E is labelled in red. Only the first 50 clustering solutions are shown for ease of visibility. Panel C shows the matrix of nodal centrality scores (each row is a node and each column is a measure) and how these are clustered in a two-cluster solution (the black and grey represent the two different clusters). Panel D shows the matrix of nodal centrality scores as well as the clusters each node was assigned to. Panel E shows a topological representation of the network, produced using the force-directed layout algorithm, where each node is coloured according to the cluster it was allocated to in panel D. (TIF) [file pone.0220061.s019.tif]

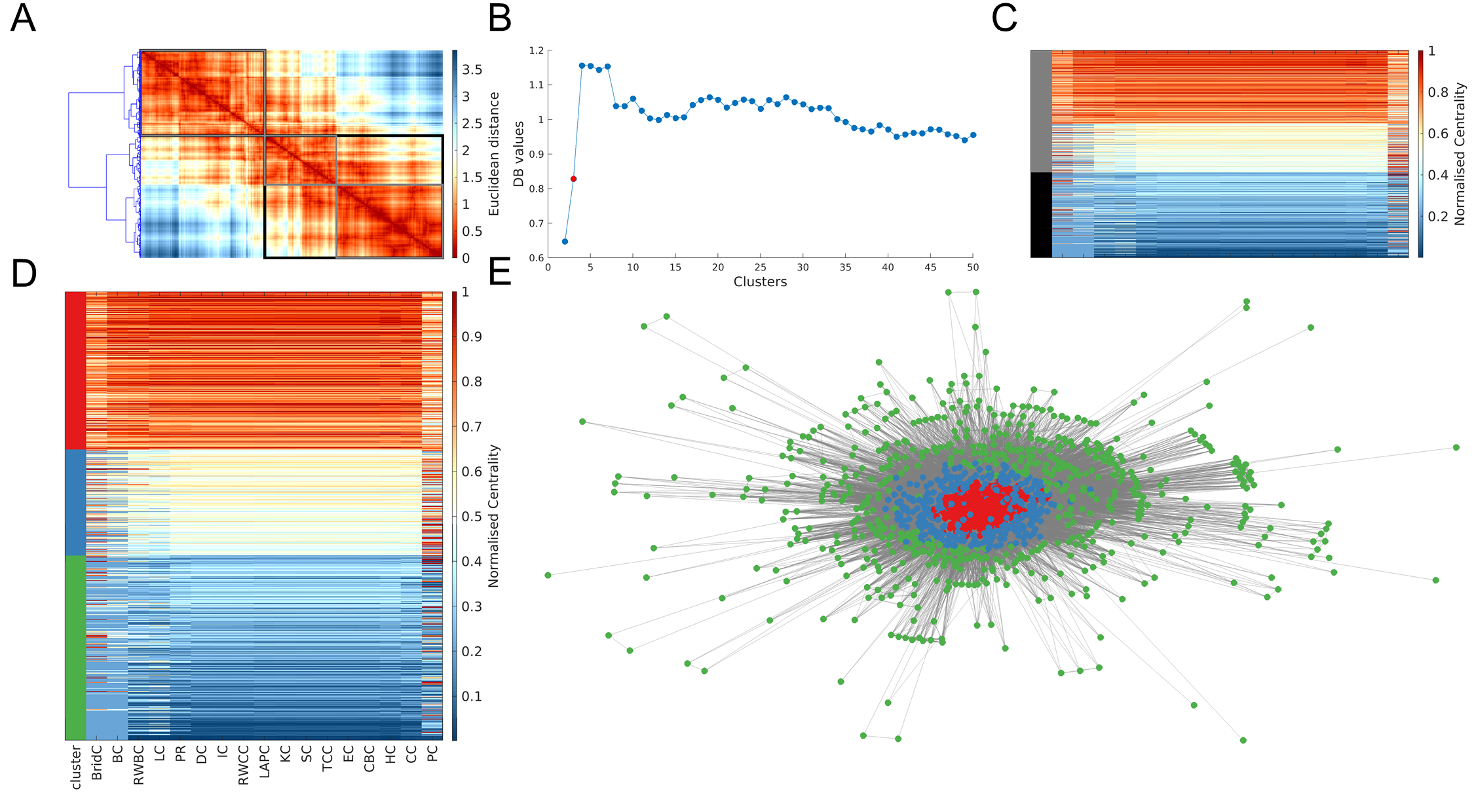

Supplement: S20 Fig — Panel A shows the dendrogram projected alongside the distance matrix of node pairs (ranks scores were normalised to be in the range 0–1 with 1 indicating the highest rank). The black and grey boxes and indicate the clusters when a two-cluster and three-cluster solution is used, respectively. Panel B displays the results for the Davies-Bouldin (DB) criterion. A lower DB value represents a better clustering solution. The solution shown in panels D and E is labelled in red. Only the first 50 clustering solutions are shown for ease of visibility. Panel C shows the matrix of nodal centrality scores (each row is a node and each column is a measure) and how these are clustered in a two-cluster solution (the black and grey represent the two different clusters). Panel D shows the matrix of nodal centrality scores as well as the clusters each node was assigned to. Panel E shows a topological representation of the network, produced using the force-directed layout algorithm, where each node is coloured according to the cluster it was allocated to in panel D. (TIF) [file pone.0220061.s020.tif]
