# Supplementary material for: Consistency and differences between centrality measures across distinct classes of networks
Source: PLoS One. 2019 Jul 26;14(7):e0220061. doi: 10.1371/journal.pone.0220061 (PMC6660088; doi:10.1371/journal.pone.0220061)
Supplement: S1 Text — (DOCX) [file pone.0220061.s021.docx]

Consistency and differences between centrality measures across distinct classes of networks

Stuart Oldham ^1*^, Ben Fulcher ^1,2^, Linden Parkes ^1^, Aurina Arnatkevic̆iūtė ^1^, Chao Suo ^1^, Alex Fornito ^1^

^1^ The Turner Institute for Brain and Mental Health, School of Psychological Sciences and Monash Biomedical Imaging, Monash University, Clayton, Victoria, Australia.

^2^ School of Physics, The University of Sydney, Sydney, New South Wales, Australia.

***Corresponding author**

E-mail: stuart.oldham@monash.edu

**Supplementary Methods**

**Centrality Definitions**

Each network is represented as an $N\times N$ adjacency matrix $A$ in which the element $A_{ij}=1$ if nodes $i$ and $j$ are connected and $A_{ij}=0$ otherwise. We denote the adjacency matrix of a weighted network $W$, where the element $W_{ij}$ encodes the weight of the edge between nodes $i$ and $j$. In the following, we present definitions of centrality measures for unweighted networks. Unless otherwise explicitly noted, these definitions were generalized to weighted networks simply substituting by $W_{ij}$ for $A_{ij}$.

**Degree/Strength (DC)**

The simplest measure of centrality is degree centrality [1], defined as the number of edges attached to a node:

$${DC}_{i}= d_{i}=\sum_{j\neq i} A_{ij}.$$

For weighted networks, we used the analogous measure of weighted degree, otherwise known as node strength $s$, which is the sum of all edge weights attached to a node,

$${DC}_{i}= s_{i}=\sum_{j\neq i} W_{ij}.$$

**H-index Centrality (HC)**

While commonly used to quantify the productivity and impact of a scientists’ work, the h-index has also been recently applied as a centrality metric in complex network analysis [2]. If $\mathcal{N}_{\geq h}\left( i \right)$ is the set of neighbours of node $i$ that have a degree equal to or greater than $h$, the h-index of node $i$can be defined as

$${HC}_{i}=\max_{1\leq h\leq:d_{i}} \min\left( \left| \mathcal{N}_{\geq h}\left( i \right) \right|,h \right),$$

where $h$ is a value between one and the degree of node $i$. Thus, h-index of a node, $i$, is defined as the maximum value $h$ for which $h$ of node $i$’s neighbours have a degree of at least $h$. When weighted networks were used, $\mathcal{N}_{\geq h}\left( i \right)$ is the set of neighbours of node $i$ that have a strength equal to or greater than $h$.

**Leverage Centrality (LC)**

Another centrality measure that considers the connections of a node’s neighbours is leverage centrality [3]. Unlikely other centrality measures, leverage centrality can assign negative values to a node, indicating that node has less connections than its neighbours. In this case, a node is said to be influenced by its neighbours. Conversely a node with positive values has more connections than its neighbours, implying that it exerts influence overs its neighbours. Leverage centrality is defined as

$${LC}_{i}=\frac{1}{d_{i}}\sum_{j \in\mathcal{N}\left( i \right)} \frac{d_{i}- d_{j}}{d_{i}+ d_{j}},$$

where $\mathcal{N}\left( i \right)$ is the set of neighbours of node $i$*.* In weighted networks the equation becomes

$${LC}_{i}=\frac{1}{d_{i}}\sum_{j \in\mathcal{N}\left( i \right)} \frac{s_{i}- s_{j}}{s_{i}+ s_{j}}.$$

**Eigenvector Centrality (EC)**

Eigenvector centrality assigns a high score to nodes that have high degree and/or have neighbours with high degree [4]. This measure is defined as the eigenvector, $v$*,* associated with the largest eigenvalue $\lambda_{1}$ of the adjacency matrix, and can be written as

$${EC}_{i}=v_{i}= \frac{1}{\lambda_{1}}\sum_{j} A_{ji}v_{j}.$$

**Katz Centrality (KC)**

In a connected network with a large, densely connected module, eigenvector centrality will assign high scores for nodes within the module and low (if not zero) scores for nodes outside the module; the measure thus becomes unsuitable for distinguishing nodes outside the module [5]. To overcome this, Katz centrality adds two parameters, $\alpha$ and $\beta$, to the definition of eigenvector centrality. The parameter $\alpha$ penalizes the contribution of distant dependencies (i.e. neighbours of neighbouring nodes) to a node’s centrality score. The parameter $\beta$ assigns a specified amount of centrality to each node, thus ensuring every node as a non-zero centrality value [6]. As Katz centrality assigns every node a small amount of centrality, this ensures that highly connected nodes in other clusters are also assigned high centrality scores. Katz centrality can be written as

$${KC}_{i}= \alpha\sum_{j} A_{ji}v_{j}+ \beta,$$

or in matrix form as

$$KC= \vec{\beta}(I- \alpha A)^{-1},$$

where $\vec{\beta}$ is a vector of size $N$ with each element equal to $\beta$ and $I$ is the identity matrix of $A$. For all analyses, $\alpha$ was set to be 10% less than the inverse of the largest eigenvalue (as typically a value close to the largest eigenvalue is used) [7] of the network and $\beta$ was set to 1.

**PageRank centrality (PR)**

With Eigenvector and Katz centrality, low degree nodes may receive a high score simply because they are connected to very high degree nodes, despite having low degree. PageRank centrality corrects for this behaviour by scaling the contribution of node $i$*'s* neighbours, $j$, to the centrality of node $i$ by the degree of $i$ [8],

$${PR}_{i}= \alpha\sum_{j} A_{ji}\frac{v_{j}}{d_{j}}+ \beta.$$

This definition can be written in matrix form as

$$PR= \vec{\beta}(I- \alpha D^{-1}A)^{-1},$$

where $D$ is a diagonal matrix and $D_{ii}$is the degree of node $i$ (in weighted networks $S$ is used instead where the diagonal $S_{ii}$ is the strength of node $i$). Note the parameters $\alpha$ and $\beta$ have the same function as in Katz centrality. $\beta$ was set to 1 and $\alpha$ was set to 0.85 for all analysis.

**Closeness Centrality (CC)**

Closeness centrality defines a node as central if it has a low average minimum path length to every other node in the network [9]. It is assumed that nodes with a short average path length to other nodes can spread or receive information in a relatively short amount of time. Since a smaller average path length indicates a more central node, the inverse is taken so that more central nodes are given higher values. This is defined by

$${CC}_{i}= \frac{N}{\sum_{j} l_{ij}},$$

where $l_{ij}$ is the shortest topological distance between nodes $i$ and $j$. For weighted networks, $l_{ij}$ was computed using the weighted shortest path (the path with the smallest edge weight sum) using the inverse of the weight matrix (as larger weight indicates greater importance in all the weighted networks used here).

**Information centrality (IC)**

This measure, also sometimes referred to as current-flow closeness centrality [10], considers all possible paths that could exist between two nodes and the overlap between these paths, and weights them per the amount of information that path contains[11]. The information in a path is defined as the inverse of the topological length of that path.

To estimate information centrality, we first define the matrix $C = (L + J)^{-1}$, where $L$ is the Laplacian of $A$ and $J$ is a $N \times N$ matrix with all elements equal to one. Information centrality is then defined as

$${IC}_{i}=\left( C_{ii}+\frac{\sum_{j} C_{jj}-2\sum_{j} C_{ij}}{N} \right)^{-1}.$$

In weighted networks $L$ is the Laplacian of $W$*.*

**Random-Walk Closeness Centrality (RWCC)**

Random-walk closeness centrality (RWCC) measures the average amount of time it takes a random-walker starting at any node in the network to reach node $i$ [12,13] and is equal to the inverse of the average mean-first passage time (MFPT) to a specific node. The MFPT can be computed from the fundamental matrix $Z$

$$Z=\left( I-P+\Pi\right)^{-1},$$

where $I$is the identity matrix, the transition matrix $P = D^{-1}A$ (or $P = S^{-1}W$ in weighted networks)*,* and $\Pi$ is a $N \times N$ matrix where each column is the vector *π* of steady state (also known as limiting) distribution probabilities of the transition matrix (such that $\Pi_{ij} = \pi_{j}$). The vector $\pi$ can be obtained by solving the system of linear equations $\pi P = \pi$ and $\sum_{i}^{N} \pi_{i}=1$. The MFPT matrix $H$ can then be defined as

$$H_{ij}= \frac{Z_{jj}-Z_{ij}}{\pi_{j}}, i\neq j.$$

The element $H_{ij}$ is the MFPT from node $i$to node $j$ [14,15]. RWCC is then simply calculated as

$${RWCC}_{i}= \frac{N}{\sum_{j} H_{ji}} .$$

**Subgraph Centrality (SC)**

Like other measures, subgraph centrality also counts the number of walks, but instead of counting walks to other nodes, this method considers closed walks (i.e. walk that begin and end at the same node) [16]. Thus, subgraph centrality measures how many subgraphs, defined by closed walks, that node belongs to, with smaller subgraphs being assigned more importance. Longer walks (and hence larger subgraphs) are penalized by weighting each walk by factor $\frac{1}{n!}$, where $n$ is the length of the walk. Thus, subgraph centrality can be computed as

$${SC}_{i}= \sum_{n=0}^{\infty} \frac{\left[ A^{n} \right]_{ii}}{n!}= \left[ e^{A} \right]_{ii}.$$

In weighted networks, the reduced adjacency matrix $S^{-\frac{1}{2}}WS^{-\frac{1}{2}}$ is used instead of $A$[17].

**Total Communicability centrality (TCC)**

Another measure which accounts for all possible walks is total communicability [18]. It is like subgraph centrality in that it considers all possible walks and walks are weighted by the inverse of the factorial of their length, but instead of just accounting for closed walks, this method also considers walks to other nodes. It is similar to information centrality in that it considers indirect routes of communication between nodes, but with walks instead of paths.

The total communicability of node $i$ can be expressed as the sum of all weighted walks to all other nodes

$${TCC}_{i}= \sum_{n=0}^{\infty} \sum_{j} \frac{\left[ A^{n} \right]_{ji}}{n!}=\sum_{j} \left[ e^{A} \right]_{ji}.$$

As with subgraph centrality, the reduced adjacency matrix $D^{-\frac{1}{2}}wD^{-\frac{1}{2}}$ is used instead of $A$ when the network is weighted.

**Laplacian centrality (LAPC)**

A node can be thought of as topologically central if its removal would impair the network in some manner. One way this can be quantified is to measure the Laplacian energy of a network

$${LAPC}_{i}= {4NW}_{2}^{C}\left( i \right)+{2NW}_{2}^{E}\left( i \right)+{2NW}_{2}^{M}\left( i \right),$$

where ${NW}_{2}^{C}\left( i \right)= \sum_{j \in\mathcal{N}\left( i \right)} W_{ij}^{2}$ which is the number of closed 2-walks involving node $i$, ${NW}_{2}^{E}\left( i \right)= \sum_{j \in\mathcal{N}\left( i \right)} \left( \sum_{k \in\mathcal{N}\left( j \right),i\neq k} W_{ij}W_{kj} \right)$ which is the number of non-closed 2-walks where $i$ is one of the end nodes, and ${NW}_{2}^{M}\left( i \right)= \frac{1}{2}\sum_{j,k \in\mathcal{N}\left( i \right)} W_{ij}W_{ik},j\neq k$ which is the number of non-closed 2-walks containing node $i$ as the middle node [19]. This definition is applicable weighted networks; in unweighted networks [20], the equation simplifies to

$${LAPC}_{i}= d_{i}^{2}+d_{i}+2\sum_{j \in\mathcal{N}\left( i \right)} d_{j}.$$

**Shortest-path Betweenness centrality (BC)**

Shortest-path Betweenness centrality defines nodes as central if they lie on many shortest-paths between other pairs of nodes [21]. The measure assumes that nodes with high betweenness act as putative information-processing bottlenecks in the network.

If $g_{jk}$ is the number of shortest paths (or geodesic paths) between nodes *p* and *q* and $g_{pq}\left( i \right)$ is the number of shortest paths between nodes $p$ and $q$ which pass through node $i$, then the betweenness centrality of node $i$ is

$${BC}_{i}= \sum_{p\neq i,p\neq q,q\neq i} \frac{g_{pq}\left( i \right)}{g_{pq}}.$$

In weighted networks the shortest path is calculated as the path with the smallest edge weight sum. As all weighted networks used here define a larger edge weight as being of more importance, the adjacency matrix was inverted prior to the shortest paths being calculated.

**Random-walk betweenness centrality (RWBC)**

The classical definition of betweenness considers only the shortest path between two nodes. However, nodes can interact through alternative routes. One way to assess such routes is to use the movements of a random-walker on a network and count how many times it passes through a given node as it travels between two others [22].

The movement of a random-walker is comparable to an electric current flowing through the network (which leads to random-walk betweenness sometimes being referred to as current flow betweenness) where each edge is a resistor and each pair of nodes is acting as a source and drain. Thus, random-walk betweenness centrality can be calculated as the average current flowing through node $i$ over all pairs of node sources $p$ and drains $q$. The currents are calculated by

$I_{i}^{\left( pq \right)}= \left\{ \begin{aligned} 1, &i=p, q \\ \frac{1}{2}\sum_{j} A_{ij}\left| T_{ip}- T_{iq}- T_{jp}+ T_{jq} \right|, &i\neq p, q \end{aligned} . \right.$

$T$ is calculated by first removing row and column *v* from the matrix $D$ and $A$, giving $D_{v}$ and $A_{v}$ respectively, and calculating the matrix $\left( D_{v} - A_{v} \right)^{-1}$. Column and row *v* are added back into this matrix with values all equal to zero to produce the matrix $T$. The random-walk betweenness centrality of node $i$ is then calculated as

$${RWBC}_{i}= \frac{\sum_{p<q} I_{i}^{\left( pq \right)}}{\frac{1}{2}N\left( N-1 \right)} .$$

In weighted networks $D$ and $A$ are substituted for $S$ and $W$ respectively. Note that this measure only considers the net-forward movement of a random-walker

**Communicability betweenness centrality (CBC)**

As previously mentioned, another way of assessing alternative routes between nodes is communicability. Communicability betweenness considers the number of walks between every pair of nodes in which a given node participates [23].

The communicability betweenness of node $i$ is

$${CBC}_{i}= \frac{1}{Ć}\sum_{p} \sum_{q} \frac{G_{piq}}{Gpq} , p\neq q,q\neq i ,$$

where $G_{piq}=\left( e^{A} \right)_{pq}-\left( e^{A+A^{'}\left( i \right)} \right)_{pq}$ is the number of walks between nodes $p$ and $q$ involving node $i$, with $A^{'}\left( i \right)$ is the adjacency matrix with all rows and columns apart from $i$ being zero, $Gpq=\left( e^{A} \right)_{pq}$ is the number of closed walks starting at $p$ and ending at $q$, and $Ć=\left( N-1 \right)^{2}-\left( N-1 \right)$ is a normalising term. As with subgraph centrality, the reduced adjacency matrix is used instead of $A$ for weighted networks.

**Bridging centrality (BridC)**

Noting that many different centrality measures are heavily influenced by nodal degree, Hwang and colleagues devised a measure known as bridging centrality, which aims to identify nodes that are central because they connect different communities/modules [24]. The measure is obtained by scaling a node’s shortest-path betweenness centrality by the nodes bridging coefficient, which is defined as

$${Bc}_{i}=\frac{d_{i}^{-1}}{\sum_{j \in\mathcal{N}\left( i \right)} d_{j}^{-1}} .$$

The coefficient quantifies the extent to which a node’s neighbours have a higher degree. Bridging centrality quantifies how many paths between highly connected nodes pass through a given node,

$${BridC}_{i}={BC}_{i}\times{Bc}_{i} .$$

**Participation coefficient (PC)**

Most real-world networks are modular [25] and nodes that play an important integrative role in the network will connect to a diverse range of modules. This distribution of a node’s connections across modules can be quantified using the participation coefficient [26]

$${PC}_{i}=1- \sum_{m=1}^{M} \left( \frac{d_{i}\left( m \right)}{d\left( i \right)} \right)^{2},$$

where $M$ is the number of modules in the network and $d_{i}\left( m \right)$ is the number of connections of node $i$ that links to nodes in module $m$. The module membership of each node was determined via consensus clustering (see below).

**Global network properties**

**Assortativity**

When nodes tend to be connected with other similar nodes, this property is known as assortativity. Commonly, this is defined in terms of node degree such that a network that is highly assortative has nodes of a similar degree connected to each other [7,27,28]. This is defined as the Pearson correlation between the degree of connected nodes and is calculated by

$$r= \frac{E^{-1}\sum_{i,j} d_{i}d_{j}-\left[ E^{-1}\sum_{i,j} \frac{d_{i}+d_{j}}{2} \right]^{2}}{E^{-1}\sum_{i,j} \frac{d_{i}^{2}+d_{j}^{2}}{2}-\left[ E^{-1}\sum_{i,j} \frac{d_{i}+d_{j}}{2} \right]^{2}},$$

where $d$ can be substituted with $s$ in weighted networks.

**Clustering**

Clustering was defined as the average number of pairs of neighbours of a node that are connected [29]

$$Cl= \frac{1}{N}\sum_{i} \frac{2t_{i}}{d_{i}\left( d_{i}-1 \right)} ,$$

where $t_{i}$ is the number of closed triangles attached to $i$. In weighted networks the clustering coefficient represents how equal the weights in the closed triangle are to the maximum edge weight in the network [30]

$${Cl}^{w}=\frac{1}{N}\sum_{i} \frac{2}{d_{i}\left( d_{i}-1 \right)} \sum_{j,k} \left( \hat{W}_{ij}\hat{W}_{kj}\hat{W}_{ik} \right)^{\frac{1}{3}},$$

where $\hat{W}_{ij}$ is where the edge weight of $i$ and $j$ have been scaled to the maximum edge weight in $W$.

**Network density**

Density is the proportion of all possible connections in a network that exist. It is defined for undirected networks as

$$\kappa= \frac{2E}{N\left( N-1 \right)},$$

where $E$ is the number of edges in the network and $N$ is the number of nodes.

**Efficiency**

The average path length $L$ of a network indicates the average length of the shortest-paths between nodes. A lower average path length indicates that less traversals have to be made to move from one node to another, in which case a network is considered to be more efficient. This is simply termed global efficiency [31], which is simply the reciprocal of $L$.

**Diffusion efficiency**

While efficiency quantifies the length of the average shortest-path, diffusion efficiency quantifies the average length of random-walks between nodes [14]

$$E_{diff}=\frac{1}{N(N-1)}\sum_{i\neq j} \frac{1}{H_{ij}} .$$

**Majorization gap**

The majorization gap quantifies the distance between an empirical network and an idealized network, called a threshold graph, in which all centrality measures rank nodes in the same way [32]. In their analysis of social networks, Schoch and Brandes [33] argued that a given metric should only be considered a measure of centrality if it preserves a property called the neighbourhood-inclusion preorder. If the neighbours of node $j$ are a subset of the neighbours of node $i$, then node $i$ is said to dominate node $j$ (a pair of nodes which share the same neighbours can be considered to have the same level of dominance over the other), and so node $i$ must have a greater or equivalent level of centrality. The neighbourhood inclusion preorder is the rank ordering of nodes in terms of these dominance relationships, such that nodes that are not dominated by any others are ranked first, and are thus more central, and nodes that are dominated by many others are ranked last (and are thus least central; e.g., S1 Fig). This preorder is complete – a dominance relationship can be expressed for every pair of nodes and thus all nodes can be ordered/ranked by their dominance relations – in a class of networks known as a threshold graphs. Such graphs are formed by adding nodes to a network, one at a time, such that the new node either connects to all existing nodes or connects to no other nodes (see S2 Fig for an example). In these networks, all centrality measures rank nodes in the same order, and this order is perfectly concordant with the neighbourhood-inclusion preorder.

Schoch and Brandes [33] argue that centrality measures will be more concordant (i.e. CMCs will be higher) in an empirical network if it is topologically similar to a comparable threshold graph. This similarity can be quantified using the *majorization gap*, which estimates the number of edges that must be rewired to transform a network into a threshold graph [32]. The majorization gap is defined as

$$Mgap=\frac{1}{2}\sum_{k=1}^{n} \max\left\{ d_{k}^{'}-ⅆ_{k},0 \right\} ,$$

where *d* is the degree sequence defined by

$$d= \left[ d_{1},d_{2}, . . ., d_{n} \right]\mathrm{with} d_{1}\geq d_{2}\geq. . . \geq d_{n} ,$$

and $d^{'}$ is the corrected conjugated sequence. For a node in position $k$ in the degree sequence, the conjugated sequence describes how many nodes before it in the degree sequence have a degree greater than or equal to $k-1$, and how many nodes following it in the degree sequence have a degree greater than or equal to $k$; formally,

$$d_{k}^{'}=\left| \left\{ i:i<k\wedge d_{\dot{i}}\geq k-1 \right\} \right|+\left| \left\{ i:i>k\wedge d_{\dot{i}}\geq k \right\} \right| \mathrm{with} 1\leq k\leq n.$$

To facilitate comparison across networks, $Mgap$ was normalised by the number of edges in the network [32].

**Modularity**

Modularity was quantified using the widely-used $Q$ metric, first proposed by Newman and Girvan [34] and defined as:

$$Q= \frac{1}{2E}\sum_{i,j} \left( A_{ij}-e_{ij} \right)\delta\left( m_{i},m_{j} \right),$$

where $E$ is the number of edges in the network, $e_{ij}= \frac{k_{i}k_{j}}{2E}$ and $\delta\left( m_{i},m_{j} \right)$ is the Kronecker delta function which is equal to one if nodes $i$ and $j$ are part of the same module and zero otherwise. For weighted networks, *E* was replaced with the total weight of unique edges in the network and *k* was replaced with the sum of all edge weights attached to a node [35]. When $Q>0$, the network exhibits greater connectivity within modules than expected by chance, under the configuration model. Modules were identified using the Louvain algorithm [36] combined with a consensus clustering procedure to address algorithmic degeneracy [37]. The Louvain algorithm was run 50 times on each network’s adjacency matrix, giving 50 partitions of the network along with an associated modularity quality score $Q$. A consensus classification matrix was computed in which each element indicated the fraction of times two nodes in the network had been assigned to the same module over the 50 iterations. This matrix was weighted by $Q$*,* such that higher quality partitions received higher weighting than lower quality partitions. A threshold of 0.4 (a value in the range 0.3-0.7 is recommend for use in Louvain clustering) [38] was applied to the matrix so that values below this threshold were set to 0. Louvain community detection was run on the thresholded matrix 50 times to produce another set of 50 partitions (approximately 50 iterations are required to produce an optimal partition). This process was repeated until the final consensus matrix resulted in node pairs either always being assigned to the same module or never being assigned to the same module [38].

**Spectral gap**

The absolute difference between the principal and second largest eigenvalues of $A$ is the spectral gap, i.e. $\left| \lambda_{1}-\lambda_{2} \right|$where $\lambda_{1}\geq\lambda_{2}\geq\ldots\geq\lambda_{N}$.. A large spectral gap is suggestive of a network having good expander properties, whereby it is both sparsely yet well connected [18,39,40]. Such networks are shown to have high correlations between walk-based centrality measures. In this paper we compute the spectral gap as the ratio $1-\frac{\lambda_{2}}{\lambda_{1}}$ (so that a larger value indicates a larger spectral gap) to provide a normalized value to allow comparison across networks [32].

**Human Brain Network Construction**

Human structural brain networks were created from the Human Connectome Project (HCP) [41]. The HCP dataset comprised diffusion-weighted MRI (1.25 mm^3^ voxel size, TE/TR = 89.5/5520ms, FOV = 210 × 180 mm, 90 directions with b = 1000, 2000, 3000 s/mm^2^, six b = 0) and T1-weighted MRI (0.7 mm3 voxel size, TR/TE = 2400/2.14ms, FOV of 224x224 mm) for 100 unrelated participants (54 males, 46 females, age range of 22-35 years) from the 500 data release. The data were acquired using a customized head coil (100 mT/m maximum gradient strength and a 32 channel head coil) on a 3T scanner located at Washington University, St Louis. All HCP data had previously gone through an extensive pre-processing pipeline [42]. Pre-processing for structural images included bias field correction, registration from native to MNI space, and segmentation of the volume into 34 cortical and seven subcortical regions for each hemisphere to produce the same 82 node parcellation as mentioned above. The HCP Diffusion MRI processing pipeline included normalization of b0 image intensity across runs, and correction for EPI susceptibility, eddy-current-induced distortions, slice dropouts, gradient-nonlinearities and subject motion.

Network nodes were defined using a recently-developed, data-driven parcellation of the cortex into 360 regions (180 per hemisphere) [43], This cortical parcellation was combined with a segmentation of seven thalamic [44,45] and three striatal [46] regions to produce a whole-brain parcellation of 380 nodes.

Diffusion images were processed using MRtrix3 [47] and the FMRIB Software Library [48]. From the corrected diffusion data, the major eigenvectors of the diffusion tensor and fibre orientation distributions (FODs) were extracted and used to conduct tractography with the Fibre Assignment by Continuous Tracking (FACT) algorithm. FACT propagates streamlines that track the trajectory of white matter tracts by following the primary direction of water diffusion at each voxel [49,50]. A total of 10 million streamlines were generated. Anatomically Constrained Tractography was employed alongside FACT using the tissue-segmented T1-weighted image to ensure that the generated streamlines were biologically accurate [51]. Dynamic seeding, where streamlines are sampled on a probabilistic basis of the relative difference between the estimated fibre density (calculated from the FOD) and current streamline reconstruction, was also employed when generating streamlines to ensure adequate sampling from across the entire brain [52]. Whole-brain tractograms were then re-weighted using Spherically Informed Filtering of Tractograms 2 (SIFT2) [52]. This algorithm adjusts streamline weights so that they more accurately represent the underlying fibre densities as estimated from the diffusion signal, and thus provide a more physiologically meaningful measure of inter-regional connectivity.

In each subject, the parcellation and tractogram were combined to produce a network map of white matter tract connectivity. The start and end points of streamlines were assigned to the closest region within a 5mm radius. This was performed for each of the 100 participants. A single group-average connectome was then created using a consistency threshold. Specifically, for each edge, we estimated the coefficient or variation across participants and retained the 5.23% most consistent edges [53]. This consistency threshold was selected based on the average density observed across individuals in the dataset.

**Surrogate Networks**

**Unconstrained surrogates**

For the unconstrained surrogate networks, we seek graphs that match only the size and density of each real-world network. A typical random network of $N$ nodes is generated by randomly allocating a set number of edges $E$ between pairs of nodes, or by forming edges between pairs of nodes with a given probability. However, such random networks are likely to be disconnected when the density is less than $\frac{\ln N}{N}$ [54]. As several networks had a density below this threshold, constructing non-fragmented surrogates (which are needed as several centrality measures assume connectedness) using standard approaches was not practically feasible for these networks. To ensure generation of a connected unconstrained/random network, we start by generating a random Minimum Spanning Tree (MST) and then add edges at random. The procedure is as follows for a network of $N$ nodes and $E$ edges:

1. Each of the $N$ nodes are listed as undiscovered and no edges are placed in the network.
2. A node is chosen at random for a random-walker to start. The random-walker can move from one node to any other (except to the node it is currently positioned on). This first node that was chosen is labelled as being discovered. The random-walker then moves to another node.
3. When the random-walk reaches an undiscovered node, that node is now labelled as discovered. An edge is added to the network connecting the previous node the random-walk was on and the current, newly discovered node.
4. The random-walk continues until all nodes are discovered. The $N-1$ edges that have been added to the network form the MST.
5. From all remaining non-existent edges, a total of $E- N - 1$ are selected uniformly at random and added in, leaving a connected random network.

This method of generating the MST ensures that the tree is generated uniformly at random [55]. Thus, when other edges are added at random, this network will have all the expected properties of a random network generated with standard algorithms. To generate weighted surrogates, edge weights of the original network were randomly assigned to edges in the connected surrogate graph.

**Constrained surrogates**

Constrained surrogates were generated using functions in the Brain Connectivity Toolbox in MATLAB [56]. The Maslov-Sneppen algorithm [57] was used for the unweighted networks to create a surrogate that preserves the number of nodes, number of edges, and degree distribution of the original network, without any fragmentation. For weighted networks, an algorithm that additionally preserves (approximately) the strength distribution of the original network was used [58].

**Supplementary results**

We ran a PCA to examine the latent structure of centrality correlations (due to the high correlations between RWCC and IC, we excluded RWCC scores from this analysis). The results revealed that the first principal component (PC1) explained 44.54-92.72% (*M* = 71.49, *SD* = 11.13) of the variance in the centrality measures in unweighted networks and 44.56-86.24% (*M* = 68.54, *SD* = 9.94) of the variance in weighted networks, which is expected giving the high collinearity between measures. While nearly all centrality measures loaded uniformly onto this component, the second and third components showed more heterogeneous loadings of measures. Betweenness-like measures would often load highly on one of these components (S4D-E Fig). Bridging centrality and the participation coefficient also emerged as loading together/uniquely onto a separate component (S4A-C Fig), which again is not surprising given these measures are the least like (both conceptually and empirically) other measures. Other types of less frequently occurring components included PageRank and leverage centrality (both measures which indicate a node is relatively more important than its neighbours) loading together (S4D-E Fig), and closeness measures loading with bridging centrality and the participation coefficient (S4F Fig).

**Table 1. General Linear Model of network properties predicting unweighted mean-within CMCs.**

| Variable | $\beta$ | *SE* | $R^{2}$ | *dfE* |
| --- | --- | --- | --- | --- |
| Model 1 | 0.869** | 0.104 | 0.505 | 205 |
| Assortativity | -0.054 | 0.028 | 0.133 |  |
| Clustering | -0.033 | 0.036 | 0.063 |  |
| Efficiency | 0.192 | 0.117 | 0.114 |  |
| Majorization gap | -0.062 | 0.067 | 0.065 |  |
| Modularity | -0.308** | 0.092 | 0.228 |  |
| Spectral gap | -0.041 | 0.055 | 0.052 |  |
| Model 2 | 0.608** | 0.070 | 0.517 | 206 |
| Assortativity | -0.039 | 0.029 | 0.095 |  |
| Clustering | -0.106** | 0.029 | 0.244 |  |
| Efficiency | 0.488** | 0.079 | 0.395 |  |
| Majorization gap | -0.095 | 0.067 | 0.097 |  |
| Spectral gap | 0.057 | 0.047 | 0.084 |  |
| Model 3 | 0.816** | 0.087 | 0.546 | 206 |
| Assortativity | -0.068** | 0.024 | 0.198 |  |
| Clustering | -0.022 | 0.034 | 0.044 |  |
| Efficiency | 0.223* | 0.112 | 0.137 |  |
| Modularity | -0.320** | 0.091 | 0.239 |  |
| Spectral gap | -0.023 | 0.051 | 0.031 |  |

Density and diffusion efficiency were not included in any model as they displayed non-linear relationships. Model 2 was run without modularity and model 3 was run without the majorization gap. * *p* < .05. ** *p* < .01.

**Table 2. General Linear Model of network properties predicting weighted mean-within CMCs.**

| Variable | $\beta$ | *SE* | $R^{2}$ | *dfE* |
| --- | --- | --- | --- | --- |
| Model 1 | 0.867** | 0.098 | 0.844 | 33 |
| Assortativity | -0.215** | 0.069 | 0.484 |  |
| Global efficiency | -0.171* | 0.073 | 0.381 |  |
| Majorization gap | -0.241 | 0.121 | 0.332 |  |
| Modularity | -0.135 | 0.155 | 0.153 |  |
| Spectral gap | 0.033 | 0.110 | 0.054 |  |
| Model 2 | 0.814** | 0.077 | 0.880 | 34 |
| Assortativity | -0.213** | 0.068 | 0.477 |  |
| Efficiency | -0.169* | 0.073 | 0.373 |  |
| Majorization gap | -0.317** | 0.083 | 0.554 |  |
| Spectral gap | 0.087 | 0.091 | 0.165 |  |
| Model 3 | 0.854** | 0.101 | 0.826 | 34 |
| Assortativity | -0.266** | 0.066 | 0.573 |  |
| Efficiency | -0.156* | 0.076 | 0.336 |  |
| Modularity | -0.359** | 0.111 | 0.489 |  |
| Spectral gap | 0.038 | 0.115 | 0.057 |  |

Density, diffusion efficiency, and clustering were not included in any model as they displayed non-linear relationships. Model 2 was run without modularity and model 3 was run without the majorization gap. * *p* < .05. ** *p* < .01.

**Supplementary references**

1. Freeman LC. Centrality in social networks conceptual clarification. Soc Networks. 1978;1: 215–239. doi:10.1016/0378-8733(78)90021-7

2. Lü L, Zhou T, Zhang Q-M, Stanley HE. The H-index of a network node and its relation to degree and coreness. Nat Commun. 2016;7: 10168. doi:10.1038/ncomms10168

3. Joyce KE, Laurienti PJ, Burdette JH, Hayasaka S. A new measure of centrality for brain networks. PLoS One. 2010;5. doi:10.1371/journal.pone.0012200

4. Phillip Bonacich. Factoring and weighting approaches to status scores and clique identification. J Math Sociol. 1972;2: 113–120.

5. Martin T, Zhang X, Newman MEJ. Localization and centrality in networks. Phys Rev E - Stat Nonlinear, Soft Matter Phys. 2014;90: 1–7. doi:10.1103/PhysRevE.90.052808

6. Katz L. A new status index derived from sociometric analysis. Psychometrika. 1953;18: 39–43.

7. Newman MEJ. Networks: An Introduction. New York: Oxford University Press; 2010.

8. Page L, Brin S, Motwani R, Winograd T. The PageRank Citation Ranking: Bringing Order to the Web. World Wide Web Internet Web Inf Syst. 1998;54: 1–17. doi:10.1.1.31.1768

9. Sabidussi G. The centrality index of a graph. Psychometrika. 1966;31: 581–603. doi:10.1007/BF02289527

10. Brandes U, Fleischer D. Centrality measures based on current flow. Lect Notes Comput Sci. 2005; 533–544. doi:10.1007/978-3-540-31856-9_44

11. Stephenson K, Zelen M. Rethinking centrality: Methods and examples. Soc Networks. 1989;11: 1–37. doi:10.1016/0378-8733(89)90016-6

12. Noh JD, Rieger H. Random Walks on Complex Networks. Phys Rev Lett. 2004;92: 1–4. doi:10.1103/PhysRevLett.92.118701

13. Blöchl F, Theis FJ, Vega-Redondo F, Fisher EON. Vertex centralities in input-output networks reveal the structure of modern economies. Phys Rev E - Stat Nonlinear, Soft Matter Phys. 2011;83: 1–9. doi:10.1103/PhysRevE.83.046127

14. Goñi J, Avena-Koenigsberger A, Velez de Mendizabal N, van den Heuvel MP, Betzel RF, Sporns O. Exploring the Morphospace of Communication Efficiency in Complex Networks. PLoS One. 2013;8. doi:10.1371/journal.pone.0058070

15. Grinstead CM, Snell JL. Introduction to Probability. Amer Mathematical Society; 2003.

16. Estrada E, Rodriguez-Velazquez J a. Subgraph Centrality in Complex Networks. Phys Rev E. 2005;71: 29. doi:10.1103/PhysRevE.71.056103

17. Crofts JJ, Higham DJ. A weighted communicability measure applied to complex brain networks. J R Soc Interface. 2009;6: 411–4. doi:10.1098/rsif.2008.0484

18. Benzi M, Klymko C. Total communicability as a centrality measure. J Complex Networks. 2013;1: 124–149. doi:10.1093/comnet/cnt007

19. Qi X, Fuller E, Wu Q, Wu Y, Zhang CQ. Laplacian centrality: A new centrality measure for weighted networks. Inf Sci (Ny). Elsevier Inc.; 2012;194: 240–253. doi:10.1016/j.ins.2011.12.027

20. Qi X, Duval RD, Christensen K, Fuller E, Spahiu A, Wu Q, et al. Terrorist Networks, Network Energy and Node Removal: A New Measure of Centrality Based on Laplacian Energy. Soc Netw. 2013;02: 19–31. doi:10.4236/sn.2013.21003

21. Freeman LC. A Set of Measures of Centrality Based on Betweenness Author. Sociometry. 1977;40: 35–41.

22. Newman MEJ. A measure of betweenness centrality based on random walks. Soc Networks. 2005;27: 39–54. doi:10.1016/j.socnet.2004.11.009

23. Estrada E, Higham DJ, Hatano N. Communicability betweenness in complex networks. Phys A Stat Mech its Appl. 2009;388: 764–774. doi:10.1016/j.physa.2008.11.011

24. Hwang W, Cho Y, Zhang A, Remanathan M. Bridging Centrality : Identifying Bridging Nodes In Scale-free Networks. Proc 14th ACM SIGKDD Int Conf Knowl Discov data Min. 2008; 336–344. doi:10.1145/1401890.1401934

25. Newman MEJ. Modularity and community structure in networks. Proc Natl Acad Sci. 2006;103: 8577–8582. doi:10.1073/pnas.0601602103

26. Guimerà R, Amaral LAN. Functional cartography of complex metabolic networks. Nature. 2005;433: 895–900. doi:10.1038/nature03288

27. Newman MEJ. Assortative Mixing in Networks. Phys Rev Lett. 2002;89: 1–4. doi:10.1103/PhysRevLett.89.208701

28. Foster JG, Foster D V., Grassberger P, Paczuski M. Edge direction and the structure of networks. Proc Natl Acad Sci. 2010;107: 10815–10820. doi:10.1073/pnas.0912671107

29. Watts DJ, Strogatz SH. Collective dynamics of’small-world’ networks. Nature. 1998;393: 440–442. doi:Doi 10.1038/30918

30. Onnela JP, Saramäki J, Kertész J, Kaski K. Intensity and coherence of motifs in weighted complex networks. Phys Rev E - Stat Nonlinear, Soft Matter Phys. 2005;71: 1–4. doi:10.1103/PhysRevE.71.065103

31. Latora V, Marchiori M. Efficient behavior of small-world networks. Phys Rev Lett. 2001;87: 198701. doi:10.1103/PhysRevLett.87.198701

32. Schoch D, Valente TW, Brandes U. Correlations among centrality indices and a class of uniquely ranked graphs. Soc Networks. Elsevier B.V.; 2017;50: 46–54. doi:10.1016/j.socnet.2017.03.010

33. Schoch D, Brandes U. Re-conceptualizing centrality in social networks. Eur J Appl Math. 2016;19: 1–15. doi:10.1017/S0956792516000401

34. Newman MEJ, Girvan M. Finding and evaluating community structure in networks. Phys Rev E. 2004;69: 026113. doi:10.1103/PhysRevE.69.026113

35. Newman MEJ. Analysis of weighted networks. Phys Rev E - Stat Physics, Plasmas, Fluids, Relat Interdiscip Top. 2004;70: 9. doi:10.1103/PhysRevE.70.056131

36. Blondel VD, Guillaume J-L, Lambiotte R, Lefebvre E. Fast unfolding of communities in large networks. J Stat Mech Theory Exp. 2008;2008: P10008. doi:10.1088/1742-5468/2008/10/P10008

37. Good BH, De Montjoye YA, Clauset A. Performance of modularity maximization in practical contexts. Phys Rev E - Stat Nonlinear, Soft Matter Phys. 2010;81: 1–19. doi:10.1103/PhysRevE.81.046106

38. Lancichinetti A, Fortunato S. Consensus clustering in complex networks. Sci Rep. 2012;2. doi:10.1038/srep00336

39. Estrada E. Spectral scaling and good expansion properties in complex networks. Europhys Lett. 2006;73: 649–655. doi:10.1209/epl/i2005-10441-3

40. Estrada E. Network robustness to targeted attacks. the interplay of expansibility and degree distribution. Eur Phys J B. 2006;52: 563–574. doi:10.1140/epjb/e2006-00330-7

41. Van Essen DC, Ugurbil K, Auerbach E, Barch D, Behrens TEJ, Bucholz R, et al. The Human Connectome Project: A data acquisition perspective. Neuroimage. 2012;62: 2222–2231. doi:10.1016/j.neuroimage.2012.02.018

42. Glasser MF, Sotiropoulos SN, Wilson JA, Coalson TS, Fischl B, Andersson JL, et al. The minimal preprocessing pipelines for the Human Connectome Project. Neuroimage. Elsevier Inc.; 2013;80: 105–124. doi:10.1016/j.neuroimage.2013.04.127

43. Glasser MF, Coalson TS, Robinson EC, Hacker CD, Harwell J, Yacoub E, et al. A multi-modal parcellation of human cerebral cortex. Nature. Nature Publishing Group; 2016;536: 171–178. doi:10.1038/nature18933

44. Behrens TEJ, Johansen-Berg H, Woolrich MW, Smith SM, Wheeler-Kingshott CAM, Boulby PA, et al. Non-invasive mapping of connections between human thalamus and cortex using diffusion imaging. Nat Neurosci. 2003;6: 750–757. doi:10.1038/nn1075

45. Behrens TEJ, Woolrich MW, Jenkinson M, Johansen-Berg H, Nunes RG, Clare S, et al. Characterization and Propagation of Uncertainty in Diffusion-Weighted MR Imaging. Magn Reson Med. 2003;50: 1077–1088. doi:10.1002/mrm.10609

46. Tziortzi AC, Haber SN, Searle GE, Tsoumpas C, Long CJ, Shotbolt P, et al. Connectivity-based functional analysis of dopamine release in the striatum using diffusion-weighted MRI and positron emission tomography. Cereb Cortex. 2014;24: 1165–1177. doi:10.1093/cercor/bhs397

47. Tournier JD, Calamante F, Connelly A. MRtrix: Diffusion tractography in crossing fiber regions. Int J Imaging Syst Technol. 2012;22: 53–66. doi:10.1002/ima.22005

48. Jenkinson M, Beckmann CF, Behrens TEJ, Woolrich MW, Smith SM. FSL. Neuroimage. 2012;62: 782–790. doi:10.1016/j.neuroimage.2011.09.015

49. Mori S, Crain BJ, Chacko VP, van Zijl PC. Three-dimensional tracking of axonal projections in the brain by magnetic resonance imaging. Ann Neurol. 1999;45: 265–269. doi:10.1002/1531-8249(199902)45:2<265::AID-ANA21>3.0.CO;2-3

50. Mori S, van Zijl PCM. Fiber tracking: principles and strategies - a technical review. NMR Biomed. 2002;15: 468–480. doi:10.1002/nbm.781

51. Smith RE, Tournier JD, Calamante F, Connelly A. Anatomically-constrained tractography: Improved diffusion MRI streamlines tractography through effective use of anatomical information. Neuroimage. Elsevier Inc.; 2012;62: 1924–1938. doi:10.1016/j.neuroimage.2012.06.005

52. Smith RE, Tournier JD, Calamante F, Connelly A. SIFT2: Enabling dense quantitative assessment of brain white matter connectivity using streamlines tractography. Neuroimage. Elsevier Inc.; 2015;119: 338–351. doi:10.1016/j.neuroimage.2015.06.092

53. Roberts JA, Perry A, Roberts G, Mitchell PB, Breakspear M. Consistency-based thresholding of the human connectome. Neuroimage. Elsevier; 2017;145: 118–129. doi:10.1016/j.neuroimage.2016.09.053

54. Barabási A-L. Network Science. United Kingdom: Cambridge University Press; 2016.

55. Wilson DB. Generating Random Spanning Trees More Quickly than the Cover Time. ACM Symposium on the Theory of Computing. 1996. pp. 296–303.

56. Rubinov M, Sporns O. Complex network measures of brain connectivity: Uses and interpretations. Neuroimage. Elsevier Inc.; 2010;52: 1059–1069. doi:10.1016/j.neuroimage.2009.10.003

57. Maslov S, Sneppen K. Specificity and Stability in Topology of Protein Networks. Science. 2002;296: 910–913. doi:10.1126/science.1065103

58. Rubinov M, Sporns O. Weight-conserving characterization of complex functional brain networks. Neuroimage. Elsevier Inc.; 2011;56: 2068–2079. doi:10.1016/j.neuroimage.2011.03.069
